# Genomic and phylogenetic characterization of ChPV2, a novel goat PV closely related to the *Xi-PV1* species infecting bovines

**DOI:** 10.1186/s12985-020-01440-9

**Published:** 2020-10-30

**Authors:** Anouk Willemsen, Alexander van den Boom, Julienne Dietz, Seval Bilge Dagalp, Firat Dogan, Ignacio G. Bravo, Anja Ehrhardt, Eric Ehrke-Schulz

**Affiliations:** 1 Chair for Virology and Microbiology, Center for Biomedical Education and Research (ZBAF), Department for Human Medicine, Faculty of Health, Witten/Herdecke University, Stockumer Strasse 10, 58453 Witten, Germany; 2 Centre National de La Recherche Scientifique (CNRS), Laboratory MIVEGEC (CNRS IRD Uni Montpellier), Montpellier, France; 3 Faculty of Veterinary Medicine, Department of Virology, Ankara University, Ankara, Turkey; 4 Faculty of Veterinary Medicine, Department of Virology, Hatay Mustafa Kemal University, Hatay, Turkey; 5 Center for Research on the Ecology and Evolution of Diseases (CREES), Montpellier, France; 6 Centre for Microbiology and Environmental Systems Science, University of Vienna, Vienna, Austria

**Keywords:** Novel goat papillomavirus, ChPV2, Genome characterization, Phylogenetic analysis

## Abstract

**Background:**

Papillomaviruses (PVs) infecting artiodactyls are very diverse, and only second in number to PVs infecting primates. PVs associated to lesions in economically important ruminant species have been isolated from cattle and sheep.

**Methods:**

Potential PV DNA from teat lesions of a Damascus goat was isolated, cloned and sequenced. The PV genome was analyzed using bioinformatics approaches to detect open reading frames and to predict potential features of encoded proteins as well as putative regulatory elements. Sequence comparison and phylogenetic analyses using the concatenated *E1E2L2L1* nucleotide and amino acid alignments was used to reveal the relationship of the new PV to the known PV diversity and its closest relevants.

**Results:**

We isolated and characterized the full-genome of novel *Capra hircus papillomavirus.* We identified the E6, E7, E1, E2, L2, L1 open reading frames with protein coding potential and putative active elements in the ChPV2 proteins and putative regulatory genome elements. Sequence similarities of *L1* and phylogenetic analyses using concatenated *E1E2L2L1* nucleotide and amino acid alignments suggest the classification as a new PV type designated ChPV2 with a phylogenetic position within the *XiPV* genus, basal to the XiPV1 species. ChPV2 is not closely related to ChPV1, the other known goat PV isolated from healthy skin, although both of them belong confidently into a clade composed of PVs infecting cervids and bovids. Interestingly, ChPV2 contains an *E6* open reading frame whereas all closely related PVs do not

**Conclusion:**

ChPV2 is a novel goat PV closely related to the Xi-PV1 species infecting bovines. Phylogenetic relationships and genome architecture of ChPV2 and closely related PV types suggest at least two independent *E6* losses within the *XiPV* clade.

## Background

Papillomaviruses (PVs) are small epitheliotropic viruses infecting mammals, reptiles, birds and fish. They are found in healthy skin and mucosa [1], benign proliferative epithelial lesions, and malignant cancers [2–4]. PVs virions are comprised of a naked capsid containing a circular, double-stranded DNA genome of approximately 8 kb organized into an upstream regulatory region (URR), an early gene region, and a late gene region [5]. The URR contains regulatory sequences for initiation of viral replication, genome maintenance and regulation of gene expression. The early region contains up to seven open reading frames (ORFs) encoding regulatory proteins (E6, E7, E5, E1, E2 and E4, nested in E2). The late region contains two ORFs encoding the capsid proteins L1 and L2. According to recent analyses, the ancestral PV genome consisted of the *E1*, *E2*, *L2* and *L1* genes, whereas the PV oncogenes (*E6*, *E7* and *E5*) were acquired later during PV evolution [6, 7]. Although the *E6* and *E7* oncogenes in PVs infecting mammals appear to have a common ancestor, several extant PV genome do lack either *E6* or *E7 *[6]*,* suggesting repeated loss of these genes [8]. PV classification is based on the nucleotide sequence similarities within *L1*, being the most conserved gene. Sequence differences of more than 10% define a new PV type if the complete genome has been cloned and sequenced. Even though most PV types share less than 60% of *L1* nucleotide identity with PVs from other genera, their assignment to species and genera requires the analysis of phylogenetic position, genome organization, biology and pathophysiology[9]. *Papillomaviridae* are divided into the *First-* and *Secondpapillomavirinae* subfamilies. *Firstpapillomavirinae* consists of only one PV, *Sparus aurata Papillomavirus* 1 (SaPV1), the only classified fish PV so far. SaPV1 is very divergent from other PVs, and has a unique genome organization containing only the URR, *E1*, *E2, L2*, and *L1* genes [10], shared by other PVs genomes isolated from other fish species (GenBank accessions MH510267, MH616908, MH617143, and MH617579). The *Secondpapillomavirinae* consist of 52 genera named after the Greek alphabet and variations thereof. Within this clade, genera can be grouped into crown-groups: four well-defined clades spanning Alpha-OmikronPVs, Beta-XiPVs, Lambda-MuPVs, Delta-ZetaPVs, an additional, ill-defined clade of PVs infecting other mammals, and a yet unclassified clade, consisting of PVs infecting birds and turtles [6].

PVs infecting cetartiodactyls are plentiful, only second in number to PVs infecting primates. They do not constitute a monophyletic group, but are scattered instead into several crown-groups. PVs infecting ruminants belong within the *Delta-*, *Xi-*, *Epsilon-*, *Dyoxi, Dyokappa-, Phi-* and *DyolambdaPV* genera within the Beta-XiPV and Delta-ZetaPV crown groups. In this manuscript we have focused on the description of a novel PV, *Capra hircus papillomavirus 2* (ChPV2), previously identified in teat-papillomas of a Damascus goat in Turkey [12]. A previously described goat PV, *Capra hircus papillomavirus 1* (ChPV1) [11] was classified as the only member of the *Phipapillomavirus,* sister taxon of a RtPV1 infecting Timor deer, and closely related to *Xipapillomavirus.* Here, we describe the genetic characterization and phylogenetic analysis of the novel goat PV ChPV2.

## Materials and methods

### Cloning and sequencing the complete ChPV2 genome

Origin of animal samples [12] and DNA extraction [13] were described before. The host animal was a Damascus goat (Shami goat) in a herd comprising about 60 animals in Hatay province in southern Turkey. Clinically, only one animal in the herd had a papilloma. According to the animals’ owners, the goats had been allowed to graze in a mountainous area, where goats and cattle used the same grazing land. The clinical sample was obtained from a teat papillomatosis case (Additional file 1: Figure S8) and sent to the laboratory at the department of Virology at Ankara University by veterinary Ali Haciömeroglu and DNA was extracted upon arrival of the specimen. No experiments with living animals were performed, nor were animals harmed or killed during any procedure related to this article.

The clinical sample was minced in phosphate-buffered saline (PBS) supplemented with streptomycin and penicillin using a scalpel and stored at − 80 °C until tested. DNA extraction from the papilloma sample was carried out according to Sambrook et al. [13].

Based on the previously published, partial *L1*-nucleotide sequence (MG523274, HTY-goat-TR2016) [12], we designed primers (5′GACTGCCCTCCTTTACAGCTT3′ and 5′GCTTTCCTGAACTTGGTAGCC3′) directed towards the edges of this fragment. The remaining part of the PV genome was amplified using Phusion DNA Polymerase kit using with standard buffer (New England Biolabs) and 100 ng of the original sample DNA as template. The resulting PCR-product was purified using the Double Pure kit (PEQLAB) according to manufacturers’ instructions and cloned into the Zero Blunt TOPO PCR Cloning plasmid (Invitrogen) according to manufacturer’s instructions. The nucleotide sequence of the cloned genome fragment was determined by primer walking from both directions using conventional Sanger sequencing (Eurofins). A ~ 2.8 kb fragment spanning the 3′ end of E2 and the 5′ end of *L2* ORF was amplified using newly designed new primers (5′GCAAATATGCTTCCCTCCATTAG3′ and 5′CTGCATAATTACACTGTCTGCAG 3′). As amplification of this fragment failed when using Phusion DNA Polymerase we amplified the missing part of the PV genome using One-Taq Polymerase 2 × Mastermix (New England Biolabs) with standard buffer and 100 ng of DNA from the original sample as a template according to manufacturer’s instructions. The resulting ~ 2.8 kb PCR fragment was gel purified using the Double Pure kit (PEQLAB) and cloned into the pGEM-T-easy plasmid (Promega) according to manufacturers’ instructions. To rule out biases resulting from the use of non-proofreading polymerase three individual clones were sequenced by primer walking (Eurofins). Resulting sequences were assembled together with the previously available genome parts and conflicts were corrected after manual inspection of sequencing results. We amplified the genomic region covering the original sequence communicated by Dogan and coworkers using the specific primers (5′TAGCTTGCTTCGCAAATTC 3′, and 5′ ATTTCGTGGCTTGCAAAGC 3′) using One-Taq Polymerase 2 × Mastermix (New England Biolabs) with standard buffer and 100 ng of DNA from the original sample as a template according to manufacturer’s instructions. The resulting PCR product was gel purified using the Double Pure kit (PEQLAB) and cloned into the pGEM-T-easy plasmid (Promega) according to manufacturers’ instructions. Three individual clones were sequenced (Eurofins) and assembled with the previously available genome parts. Conflicts with the previously published sequence from Dogan and coworkers were corrected after comparative alignment and manual inspection. Finally we performed rolling circle amplification (RCA) using the TempliPhi amplification kit (GE healthcare). Resulting RCA products were subjected to MfeI restriction digest to the linearize PV genomes into single genome copies. The resulting restriction fragments of ~ 7 kb was gel-purified and cloned into pShV plasmid. A positive clone was sequenced by primer walking and the previously assembled sequence was confirmed.

### Sequence analysis

ORF analysis was performed using the ORF Finder tool implemented in SnapGene (https://www.snapgene.com/) and reviewed manually by comparative alignment to closely related PV genotypes. Potential splicing patterns for the E1^E4 ORF were predicted using the Softberry Fsplice program (https://www.softberry.com/berry.phtml?topic=fsplice&group=programs&subgroup=gfind) with the *Capra hircus* genome as a reference. Sequence similarities were calculated based on the single gene alignments. The molecular weight of the putative proteins was calculated using the ExPASy (Expert Protein Analysis System) Compute pI/Mw tool (https://au.expasy.org/tools/pi_tool.html) [14]. Protein motifs and domains were identified manually or predicted using prosite tool (https://prosite.expasy.org/) [15, 16]. TATA Box, pA signals as well as E2 and E1 binding sites were identified manually. Potential E2 binding sites were reconfirmed by screening the TRANSFAC Database [17] using the Match tool (https://gene-regulation.com/pub/programs.html#match) [18] as described previously [19].

### Phylogenetic analysis

We collected 376 full-length PV genomes from the PaVE database (pave.niaid.nih.gov, accessed 28 May 2019) and the ChPV2 genome was added to this data set (Additional file 1: Table S6). The *E1*, *E2*, *L2* and *L1* genes were extracted from the collected genomes. Genes were aligned individually at the amino acid level using MAFFT v.7.310 [20], corrected manually, and backtranslated to nucleotides using PAL2NAL v.14 [21]. The alignments were filtered using Gblocks v.0.91b [22].

The full 377 PV data set contained recombinant PVs infecting Cetaceans [23–25], known to have undergone a recombination event between the early and the late gene regions. Therefore initial tree construction was performed on the concatenated *E1E2* and *L2L1* alignments separately. Maximum Likelihood (ML) phylogenetic inference was done using RAxML v8.2.11 [26], under the GTR + Γ4 model for the nucleotide alignments using six partitions (three for each gene corresponding to each codon position), or under the LG + Γ model for the amino acid alignment using two partitions (one for each gene), and using 1000 bootstrap replicates. The trees were rooted using the SaPV1 sequence.

Subsequently, the recombinant and unresolved taxa were removed from the full data set, leaving us with a reduced data set of 324 PVs. The individual genes were again aligned and filtered as described above. The concatenated *E1E2*, *L2L1*, and *E1E2L2L1* alignments were used to construct ML trees as described above. For the *E1E2L2L1* alignment, twelve partitions were used at the nucleotide level, and four partitions were used at the amino acid level. The trees were rooted using the SaPV1 sequence.

Based on the constructed trees, the close relatives of ChPV2 were extracted from the full data set, leaving us with a reduced data set of 17 PVs. Besides *E1*, *E2*, *L2*, and *L1*, the *E6* and *E7* genes were extracted from these selected genomes. The individual genes were aligned without Gblocks filtering. The *E6*, *E7*, *E1*, *E2*, *L2*, *L1*, and the concatenated *E1E2L2L1* alignments were used to construct ML trees for this reduced data set as described above. For the individual gene alignments, three partitions were used at the nucleotide level, and no partitions were used at the amino acid level. For the *E1E2L2L1* alignment, twelve partitions were used at the nucleotide level, and four partitions were used at the amino acid level. Possible rogue taxa were identified with an algorithm implemented in RAxML. For the individual gene trees, majority rule consensus trees were constructed, and subsequently used for constructing a supernetwork using Splits Tree 4 [27].

## Results

### Cloning and sequence assembly PV genome

To isolate the complete genomic DNA of a PV identified in DNA samples from teat papillomas of a Damascus goat in Turkey [12], we performed long range PCR using a primer set directed towards the boarders of the previously published, partial *L1*-nucleotide sequence (MG523274, HTY-goat-TR2016). The resulting PCR-product was cloned and sequenced by primer walking. Based on the resulting sequence assembly we finally amplified the genome region spanning the partial *L1* sequences published by Dogan and coworkers *2*018 from *the* original DNA sample. The resulting PCR fragment was cloned, sequenced and assembled with the previously available genome parts. Conflicts with the previously published sequence were corrected after manual inspection of a comparative alignment. MfeI restriction digest of rolling circle amplified (RCA) concatenated linearized PV genome copies generated full genome fragments that were cloned respectively. Sequencing confirmed the previously assembled sequence. Plasmids are available from Eric Ehrke-Schulz upon request. The nucleotide sequence of the ChPV2 genome is accessible in GenBank under accession number MN148899.

### Genome characterization

The genome of ChPV2 spans 7295 bp with an average GC content of 43% and has the typical organization of a PV genome, containing an upstream regulatory region (URR), an early gene region and a late gene region (Additional file 1: Figure S1, Table S1). The early region contains five putative partially overlapping ORFs, *E6* (417 bp), *E7* (297 bp), *E1* (1809 bp), *E2* (1293 bp), and *E4* nested within *E2* (336 bp). Splice site prediction suggested three different potential *E1^E4* splice patterns, with nucleotide position 712 as donor and positions 3052, 3088 or 3226 as putative acceptors (Additional file 1: Table S1). Nucleotide and amino acid sequence similarities of ChPV2 genes to their counterparts of closely related PV types were determined based on single gene alignments of ChPV2 and 16 closely related PV types (Additional file 1: Table S2 and Table S3).

### Prediction of potential protein features

Within the translated ORFs of ChPV2 several protein domains/motifs were predicted (Additional file 1: Table S4). ChPV2-E6 contains two zinc-binding motifs (C-X_2_-C-X_28_-C-X_2_-C), but it does not seem to contain a standard PDZ-binding motif. ChPV2-E7 contains a casein kinase II phosphorylation site followed by a retinoblastoma protein (pRB) binding motif (LXCXE) and a zinc binding motif (C-X_2_-C-X_28_-C-X_2_-C). In ChPV2-E1 a SF3 helicase 1 domain was identified. ChPV2-E2 contains a DNA binding Helix (GCANTLKCFRRRTSHSHPHK). The late proteins ChPV2-L2 and ChPV2-L1 contain lysine and arginine rich nuclear localization signals (RKFKRKTK) (Additional file 1: Table S4).

### Prediction of potential regulatory elements

A number of non-coding regulatory elements were found throughout the ChPV2 genome (Additional file 1: Table S5, Additional file 1: Figure S2). Within the URR a potential TATA box (TATAAA) is located from bp 7273 to 7277, 18 bp upstream of the E6 start codon. Upstream of the TATA Box, three potential E2 binding sites (E2BS), (ACC-N_6_-GGT) are located at nucleotide positions 7164–7175, 7242–7253, and 7258–7269. A potential E1 binding site (E1BS), (GTAGTTGTTGTTAACAACAAT) is located between the first and second E2BS. Two imperfect E2BS* (ACT-N_6_-GGT, bp 455 to bp 466) and (ACC-N_6_-GTG, bp 481 to bp 492) are located within the E7 ORF, 64 bp upstream of a potential TATA box (TATAA) at nucleotide positions 555 to 559, and could constitute the late promoter. Another potential E2BS (ACC-N_6_-GGT, positions 6789–6796) is located close to the 3′ end of the *L1* ORF. An early polyadenylation signal (pA, AATAAA) is located downstream of the *E2* ORF (bp 3749 to bp 3754) and a late pA is located downstream of the *L1* ORF (bp 6920 to bp 6925).

### Phylogenetic analysis

In order to assess the phylogenetic relationships of ChPV2, we collected the available 376 full-length PV genomes from the PaVE database (pave.niaid.nih.gov, accessed 28 May 2019) (Additional file 1: Table S6). First, we constructed ML phylogenetic trees of the concatenated early (*E1E2*) and late (*L2L1*) gene sequences at the nucleotide (Additional file 1: Figure S2) and amino acid (Additional file 1: Figure S3) levels. Based on these four trees, we observe that ChPV2 clusters within the *Beta-XiPV* crown group, and is closely related to *XiPVs* infecting cetartiodactyles. The position of ChPV2 is well-supported (bootstrap support of 97 to 100) and is basal to *XiPVs, 1* species (infecting bovines).

**Fig. 1:**
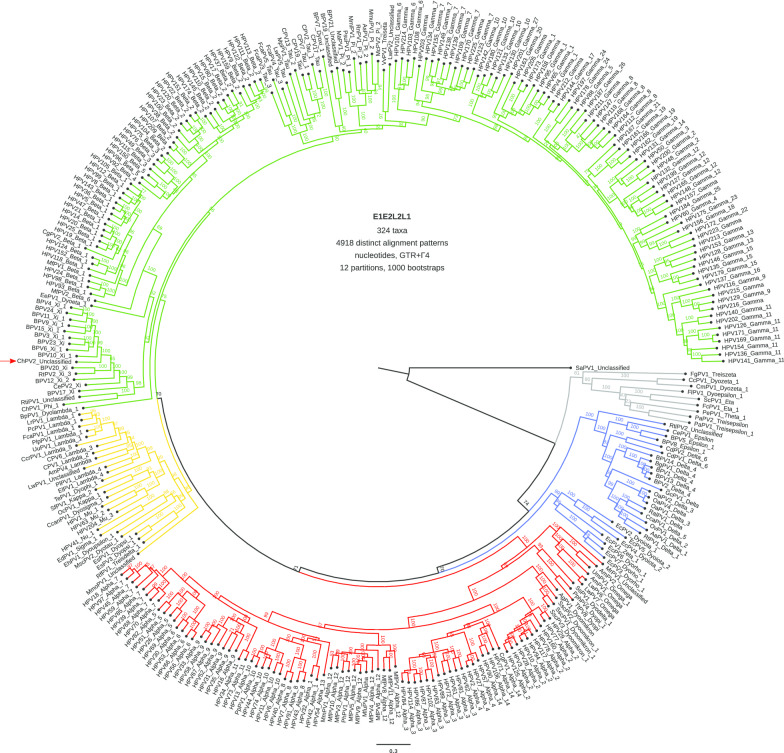
Maximum likelihood phylogenetic tree of the concatenated E1E2L2L1 nucleotide alignments of 324 PVs. Clade color codes highlight the different PV groups: red, Alpha-OmikronPVs; green, Beta-XiPVs; yellow, Lambda-MuPVs; blue, Delta-ZetaPVs; and gray, a yet unclassified clade consisting of PVs infecting birds and turtles. The position of ChPV2 is indicated with a red arrow. Values on branches correspond to ML bootstrap support values

After removing the recombinant and unresolved taxa, ML phylogenetic trees were constructed of the concatenated *E1E2* and *L2L1* alignments (Additional file 1: Figure S4 and Additional file 1: Figure S5)*,* as well as of the concatenated *E1E2L2L1* alignments at the nucleotide (Fig. 1) and amino acid (Additional file 1: Figure S6) levels. The position of ChPV2 did not change and remained well supported. Interestingly, ChPV2 does not cluster with the previously described ChPV1, retrieved from the healthy skin of a goat, and classified as *PhiPV1 (11)*. ChPV1 is the sister taxon to the Timor deer RtimPV1, and both are basal to *XiPVs*. The nucleotide sequence of the *ChPV2-L1* gene shows higher similarities to *L1* genes of other *XiPVs* and to RtimPV1 ranging from 58.3 to 71.8%, than to ChPV1 *L1*, where the nucleotide similarity is 55.3% (Table 1).

**Table 1:** L1 gene nucleotide (nt) similarity matrix of ChPV2 and closely related PV types, yet unclassified PV types are marked with a question sign

% L1 nt similarity	ChPV2?	BPV3 XiPV	BPV4 XiPV	BPV6 XiPV	BPV9 XiPV	BPV10 XiPV	BPV11 XiPV	BPV12 XiPV	BPV15 XiPV	BPV17 XiPV	BPV20 XiPV	BPV23 XiPV	BPV24 XiPV	CePV2 XiPV	ChPV1 PhiPV	RtPV2 XiPV	RtiPV1?
ChPV2 ?		69.7	70.1	72.0	70.2	71.3	70.3	66.5	67.5	58.3	64.7	69.2	69.6	63.8	55.3	67.5	59.9
BPV3 XiPV	69.7		72.5	72.0	73.0	70.1	72.0	67.1	72.2	60.2	64.8	78.3	70.3	65.4	56.6	65.3	59.2
BPV4 XiPV	70.1	72.5		71.5	73.3	69.7	77.6	66.2	72.1	60.2	65.4	72.8	77.4	64.2	56.2	66.3	60.9
BPV6 XiPV	72.0	72.0	71.5		71.4	69.2	71.2	67.0	70.0	60.8	66.5	74.1	71.2	64.4	57.6	66.9	60.4
BPV9 XiPV	70.2	73.0	73.3	71.4		70.9	74.3	65.8	74.5	59.7	66.6	73.3	72.6	63.5	57.2	66.7	59.6
BPV10 XiPV	71.3	70.1	69.7	69.2	70.9		69.9	65.6	69.6	58.3	66.5	72.5	69.2	63.9	56.8	67.1	59.9
BPV11 XiPV	70.3	72.0	77.6	71.2	74.3	69.9		65.3	73.6	60.4	65.2	73.7	75.7	64.0	57.0	65.9	60.9
BPV12 XiPV	66.5	67.1	66.2	67.0	65.8	65.6	65.3		66.6	61.0	67.1	67.0	67.1	65.0	56.2	69.0	60.1
BPV15 XiPV	67.5	72.2	72.1	70.0	74.5	69.6	73.6	66.6		60.8	66.2	72.2	73.8	64.0	56.5	65.3	59.8
BPV17 XiPV	58.3	60.2	60.2	60.8	59.7	58.3	60.4	61.0	60.8		60.4	59.2	60.4	58.6	53.7	60.2	58.1
BPV20 XiPV	64.7	64.8	65.4	66.5	66.6	66.5	65.2	67.1	66.2	60.4		64.4	65.2	63.7	56.8	73.4	59.9
BPV23 XiPV	69.2	78.3	72.8	74.1	73.3	72.5	73.7	67.0	72.2	59.2	64.4		72.1	65.3	56.7	65.5	59.7
BPV24 XiPV	69.6	70.3	77.4	71.2	72.6	69.2	75.7	67.1	73.8	60.4	65.2	72.1		62.2	57.2	65.9	59.8
CePV2 XiPV	63.8	65.4	64.2	64.4	63.5	63.9	64.0	65.0	64.0	58.6	63.7	65.3	62.2		55.5	64.8	58.5
ChPV1 PhiPV	55.3	56.6	56.2	57.6	57.2	56.8	57.0	56.2	56.5	53.7	56.8	56.7	57.2	55.5		57.1	60.3
RtPV2 XiPV	67.5	65.3	66.3	66.9	66.7	67.1	65.9	69.0	65.3	60.2	73.4	65.5	65.9	64.8	57.1		60.0
RtiPV1?	59.9	59.2	60.9	60.4	59.6	59.9	60.9	60.1	59.8	58.1	59.9	59.7	59.8	58.5	60.3	60.0	

**Fig. 2:**
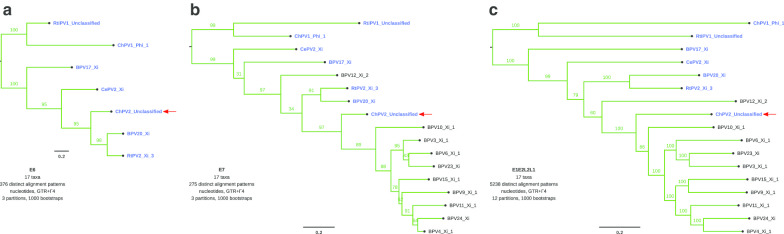
Maximum likelihood phylogenetic trees of E6 (**a**), E7 (**b**) and the concatenated E1E2L2L1 (**c** nucleotide alignments of 17 PVs. The taxa highlighted in blue contain both the E6 and E7 oncogenes in their genomes, while the other taxa contain only E7. The position of ChPV2 is indicated with a red arrow. Values on branches correspond to ML bootstrap support values

Subsequently we constructed ML phylogenetic trees only with the close relatives of ChPV1, including also the *E6* and *E7* oncogene alignments, as well as the concatenated *E1E2L2L1* alignments on the nucleotide (Fig. 2) and amino acid levels (Additional file 1: Figure S7). Not all close relatives of ChPV2 contain the *E6* oncogene in their genomes (Fig. 2 and Additional file 1: Figure S7). These PVs lacking the E6 protein encode instead for a small hydrophobic protein (between 40 and 75 amino acids) annotated upstream, named *E10* [8]. Interestingly, all PV genomes containing only *E6* cluster together, except for BPV12 that clusters close to PVs containing both *E6* and *E7* in their genomes (Fig. 2 and Additional file 1: Figure S7).

**Fig. 3:**
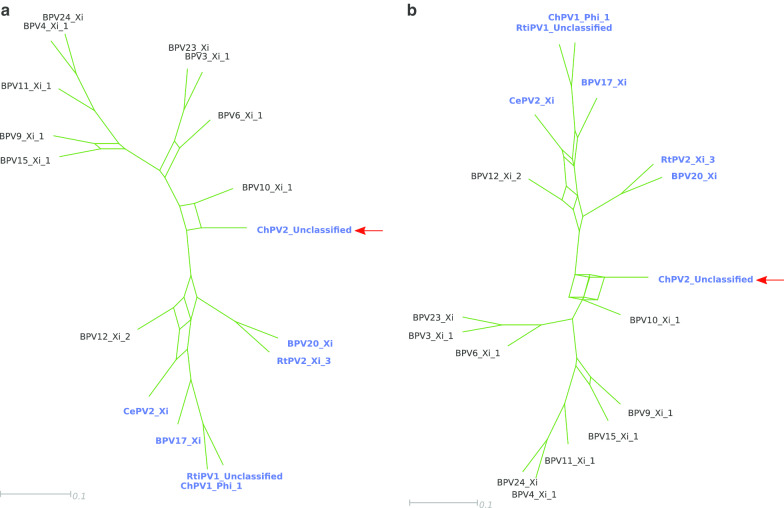
Supernetwork constructed from the majority rule consensus trees of the E6, E7, E1, E2, L2, and L1 genes, at the nucleotide (**a**) and amino acid (**b**) levels of 17 PVs. The taxa highlighted in blue contain both the E6 and E7 oncogenes in their genomes, while the other taxa contain only E7. The position of ChPV2 is indicated with a red arrow

To complement the current view, the individual gene trees (*E6*, *E7*, *E1*, *E2*, *L1*, and *L1*) were combined to construct a phylogenetic supernetwork (Fig. 3). Our results show few conflicting topologies between the trees, and confirmed that ChPV2 is closely related to the *Xipapillomavirus 1* species, and in particular to BPV10. Moreover, the positioning of BPV12 does not seem to change much between trees and remains within the clade of PVs containing both *E6* and *E7*.

## Discussion

Here we present the genomic and phylogenetic characterization of ChPV2 isolated from a teat wart of a Damascus goat from Turkey. Its genome shows the typical organization of PV genomes and contains seven putative genes *E6, E7, E1, E2 E4* as well as *L2* and *L1*. Splice site prediction suggested three different potential *E1^E4* splice patterns, one out them encoding for a very small potential protein of only 33 aa that seems unlikely to be expressed. Further experimental evidence is needed to verify transcription and potential splicing and translation of *E1^E4* mRNAs.

The ability of PVs to trigger cell proliferation is related to specific activities of the oncogenes the PV genome contains. In oncogenic human papillomaviruses (HPVs) the major oncoproteins are E6 and E7. E6 proteins from oncogenic HPVs interact with a series of host cell proteins through two zinc finger domains containing the CXXC motif [28–32], that binds LXXLL motifs in their cellular counterparts. The paradigm of these interactions is the recruitment of cellular E3 ubiquitin ligase (E6AP) to target p53 to proteasomal degradation [33, 34]. Again in oncogenic HPVs E6 a short C-terminal PDZ-binding motif (X-T/S-X-V/L) further facilitates interactions with proteins containing PDZ domains [30, 31, 35–37], promoting for instance the induction of epithelial hyperplasia anchorage-independent growth and tumorigenic potential [36, 38], but it is not necessary for maintenance of the PV genome [39]. In the novel ChPV2, the E6 protein contains two zinc binding motifs but no PDZ binding motif, suggesting low transforming potential of this protein.

In oncogenic HPVs, E7 interacts with members of the retinoblastoma protein family, (pRB), through conserved regions that contain pRB binding motif (LXCXE) followed by a casein kinase II (CKII) phosphorylation site and a zinc binding domain [40, 41]. This interaction leads to the dissociation of pRB and E2F transcription factor, thus promoting cell cycle progression that can eventually lead to uncontrolled cell growth and to the development of proliferative lesions. In ChPV2 E7 all components described to be necessary for effective binding of pRB, seem to be present, suggesting effective pRB binding. Whether the presence of these motifs contribute to proliferation of the ChPV2 associated teat warts needs further experimental evidence.

The presence and positions of regulatory elements within the ChPV2-URR and early genes differ from the previously described ChPV1 [11] (Additional file 1: Table S5, Figure S2). The ChPV2-URR contains a TATA box upstream of the *E6* start codon. Upstream of the TATA box a potential E1BS was identified, that is accompanied by three E2BSs, of which one E2BS is located 25 bp upstream and two sites located 23 and 38 bp downstream of the E1BS. The equidistant proximity of the two E2BS flanking the E1BS, suggests a functional role for ChPV2 genome replication. Whether the third E2 binding site contributes to replication control or transcriptional regulation of the ChPV2 early promotor remains unclear and needs further experimental evidence. It can only be speculated, whether the fourth potential E2 binding site at the 3′ end of the L1 ORF is functionally relevant. Additionally, two imperfect E2BS were identified in close proximity towards each other within the *E7* ORF. Probably they represent an enhancer element of the ChPV2 late promoter as they are located closely upstream of a potential TATA box representing the core element of the ChPV2 late promoter. Because of one mismatch to these E2BS consensus sequence, it remains to be determined experimentally whether these elements are functional or bind E2 with less affinity than the perfect E2BS within the URR.

ChPV2 clusters within the *Beta-XiPV* crown group, being confidently basal to *XiPV1*. *XiPV1* have been isolated from papillomas of the upper alimentary canal [42] and cutaneous papillomas of cattle from Great Britain [43] cutaneous warts of cattle from Japan and squamous papilloma lesions on cattle teats from Japan [44, 45] as well as cutaneaous papillomatous lesion of cows in Brazil [46, 47]. Apart from a distinct geographical occurrence and different host species, ChPV2 shares its cutaneous localization and teat involvement with the closely related *XiPV1,* further supporting its phylogenetic position. The nucleotide sequence of the ChPV2 *L1* gene (Table 1) shows nucleotide identity to *L1* genes of other *XiPVs* ranging between 58.3% and 71.8%. According to the conventions for naming and grouping of PVs, these findings supports the definition of ChPV2 as a new PV type as member of *XiPV1*.

Interestingly, the only other known goat PV, ChPV1, isolated from healthy skin [11], is not closely related to ChPV2. The position of the sister taxa ChPV1 and RtimPV1 is well supported and consistently basal to XiPVs. However, *L1* nucleotide identity values are borderline for the current standards, and the taxonomic relationship of these ChPV1 and RtimPV1 with respect to XiPVs has to be elucidated by the ICTV.

The fact that the ChPV2 infected goat shared space with cattle, opens the door to speculate about virus circulation between species. Although ChPV2 is closely related to bovine PVs, sequence similarities between ChPV2 and its closest relatives are probably too small to claim, that ChPV2 arose from an interspecies transmission event. We believe that, ChPV2 and/or closely related Xi PVs, that infect bovids, ovids and cervids might rather represent broad spectrum PVs, that could be able to infect different host species. Therefore, ChPV2 and closely related PVs might rather mimic the case of BPV1, which can infect cattle, sheep, several deer species, horses, zebras and tapirs. Currently, we do not know how prevalent this virus is among goats, or whether it is present in cattle alongside. Further studies are necessary to investigate the relative prevalence of the virus in the different species, as well as the efficiency of transmission within and between species to allow for differentiation between the broad spectrum PV and the interspecies transmission hypothesis.

Both the *E6* and *E7* oncogenes in PVs infecting mammals appear to have a common ancestor [6]. It has been suggested previously that *E6* may have been lost two separate times within the *XiPV* clade [8]. Among the closest relatives of ChPV2 all PV genomes containing only *E7* cluster together, except for BPV12 that clusters within PVs containing both *E6* and *E7* (Fig. 2 and Additional file 1: Figure S7), supporting the repeated loss of *E6* hypothesis [6, 8]. However, it is possible that the separate *E6* and *E7*, and concatenated *E1E2L2L1* gene trees do not accurately describe the evolutionary history of these PVs. Nonetheless, we did not identify any rogue taxa for the constructed trees.

## Conclusion

Broadening the spectrum of known PVs infecting artiodactyls will probably improve future phylogenetic inference allowing for a more detailed resolution and interpretation of the phylogenetic relationships of the artiodactyl PVs within *Phi- and Xi-PVs.*

## Supplementary information

**Additional file 1: Figure S1.** Genome organization of ChPV2; Upstream regulatory region, yellow; early genes, green late genes, blue, potential E4 gene and potential E1^E4 splicing patterns, ochre. **Figure S2.** Presence and position of putative regulatory elements within URR and early gene region of ChPV1 and ChPV2, E1- binding sites (E1BS) are marked by yellow boxes, E2- binding sites (E2BS) perfectly matching the E2BS consensus sequence are marked by red boxes, E2BS with 1 nucleotide mismatch to the E2BS consensus sequence are marked in orange. nucleotide positions of early genes and regulatory elements are depicted below or above the respective element. **Figure S3.** Maximum likelihood phylogenetic trees of the concatenated E1E2 and concatenated L2L1 nucleotide alignments of 377 PVs. Clade color codes highlight the different PV groups and recombinant taxa: red, Alpha-OmikronPVs; green, Beta-XiPVs; yellow, Lambda-MuPVs; blue, Delta-ZetaPVs; gray, a yet unclassified clade consisting of PVs infecting birds and turtles; aqua-blue, a yet unclassified clade consisting of PVs infecting Caniformia; orange, known recombinant PVs infecting Cetaceans; and pink, possible recombinant PVs. The position of ChPV2 is indicated with a red arrow. Values on branches correspond to ML bootstrap support values. **Figure S4.** Maximum likelihood phylogenetic trees of the concatenated E1E2 and concatenated L2L1 nucleotide alignments of 324 PVs. Clade color codes highlight the different PV groups: red, Alpha-OmikronPVs; green, Beta-XiPVs; yellow, Lambda-MuPVs; blue, Delta-ZetaPVs; and gray, a yet unclassified clade consisting of PVs infecting birds and turtles. The position of ChPV2 is indicated with a red arrow. Values on branches correspond to ML bootstrap support values. **Figure S5.** Maximum likelihood phylogenetic trees of the concatenated E1E2 and concatenated L2L1 amino acid alignments of 324 PVs. Clade color codes highlight the different PV groups: red, Alpha-OmikronPVs; green, Beta-XiPVs; yellow, Lambda-MuPVs; blue, Delta-ZetaPVs; and gray, a yet unclassified clade consisting of PVs infecting birds and turtles. The position of ChPV2 is indicated with a red arrow. Values on branches correspond to ML bootstrap support values. **Figure S6. **Maximum likelihood phylogenetic tree of the concatenated E1E2L2L1 amino acid alignments of 324 PVs. Clade color codes highlight the different PV groups: red, Alpha-OmikronPVs; green, Beta-XiPVs; yellow, Lambda-MuPVs; blue, Delta-ZetaPVs; and gray, a yet unclassified clade consisting of PVs infecting birds and turtles. The position of ChPV2 is indicated with a red arrow. Values on branches correspond to ML bootstrap support values. **Figure S7. **Maximum likelihood phylogenetic trees of E6 (A), E7 (B) and the concatenated E1E2L2L1 (C) amino acid alignments of 17 PVs. The taxa highlighted in blue contain both the E6 and E7 oncogenes in their genomes, while the other taxa contain only E7. The position of ChPV2 is indicated with a red arrow. Values on branches correspond to ML bootstrap support values. **Figure S8.** Macroscopic view of teat papilloma of a Damascus (Shami) goat, taken from (12). **Table S1.** Genome organization of ChPV2 presenting orf positions, orf length, GC content and amino acid content of encoded potential proteins and their molecular weight. **Table S2.** Nucleotide (nt) identity of ChPV2 genes with the respective genes of closely related PV types. **Table S3.** Amino acid (aa) identity of ChPV2 genes with the respective genes of closely related PV types. **Table S4.** Potential motifs and domains identified in amino acid sequences of ChPV2 proteins. **Table S5.** Potential regulatory elements identified throughout the ChPV2 genome, elements marked with a * have a mismatch to the published consensus sequence for the respective element. **Table S6.** List of PV genomes collected from PaVE (pave.niaid.nih.gov) plus ChPV2.

## Data Availability

The nucleotide sequence of the ChPV2 genome is accessible in GeneBank under accession number MN148899 under https://www.ncbi.nlm.nih.gov/nuccore/MN148899: The authors confirm all supporting data, code and protocols have been provided within the article or through supplementary data files.

## Abbreviations

PVs: Papillomaviruses
ORFs: Open reading frames
RCA: Rolling circle amplification
ML: Maximum likelihood
URR: Upstream regulatory region
pRB: Retinoblastoma protein
E2BS: E2 binding site
E1BS: E1 binding site
pA: Polyadenylation signal
HPV: Human papillomavirus
E6AP: E3 ubiquitin ligase
CKII: Casein kinase II

## References

[1] Antonsson A, Hansson BG (2002). Healthy skin of many animal species harbors papillomaviruses which are closely related to their human counterparts. J Virol.

[2] Nindl I, Gottschling M, Stockfleth E (2007). Human papillomaviruses and non-melanoma skin cancer: basic virology and clinical manifestations. Dis Mark.

[3] Hubbers CU, Akgul B (2015). HPV and cancer of the oral cavity. Virulence.

[4] zur Hausen H (2002). Papillomaviruses and cancer: from basic studies to clinical application. Nat Rev Cancer.

[5] Bravo IG, Felez-Sanchez M (2015). Papillomaviruses: viral evolution, cancer and evolutionary medicine. Evol Med Public Health.

[6] Willemsen A, Bravo IG (2019). Origin and evolution of papillomavirus (onco)genes and genomes. Philos Trans R Soc Lond B Biol Sci.

[7] Willemsen A, Felez-Sanchez M, Bravo IG (2019). Genome plasticity in papillomaviruses and de novo emergence of E5 oncogenes. Genome Biol Evol.

[8] Van Doorslaer K, McBride AA (2016). Molecular archeological evidence in support of the repeated loss of a papillomavirus gene. Sci Rep.

[9] Bernard HU, Burk RD, Chen Z, van Doorslaer K, zur Hausen H, de Villiers EM (2010). Classification of papillomaviruses (PVs) based on 189 PV types and proposal of taxonomic amendments. Virology.

[10] Lopez-Bueno A, Mavian C, Labella AM, Castro D, Borrego JJ, Alcami A (2016). Concurrence of iridovirus, polyomavirus, and a unique member of a new group of fish papillomaviruses in lymphocystis disease-affected gilthead sea bream. J Virol.

[11] Van Doorslaer K, Rector A, Vos P, Van Ranst M (2006). Genetic characterization of the *Capra hircus* papillomavirus: a novel close-to-root artiodactyl papillomavirus. Virus Res.

[12] Dogan F, Dorttas SD, Bilge Dagalp S, Ataseven VS, Alkan F (2018). A teat papillomatosis case in a Damascus goat (Shami goat) in Hatay Province, Turkey: a new putative papillomavirus?. Arch Virol.

[13] Maniatis T, Fritsch EF, Sambrook J (2001). Molecular cloning: a laboratory manual.

[14] Gasteiger E, Hoogland C, Gattiker A, Duvaud S, Wilkins MR, Appel RD, Walker JM (2005). Protein identification and analysis tools on the ExPASy server. The proteomics protocols handbook.

[15] Sigrist CJ, de Castro E, Cerutti L, Cuche BA, Hulo N, Bridge A (2013). New and continuing developments at PROSITE. Nucleic Acids Res.

[16] Sigrist CJ, Cerutti L, Hulo N, Gattiker A, Falquet L, Pagni M (2002). PROSITE: a documented database using patterns and profiles as motif descriptors. Brief Bioinform.

[17] Wingender E, Dietze P, Karas H, Knuppel R (1996). TRANSFAC: a database on transcription factors and their DNA binding sites. Nucleic Acids Res.

[18] Kel AE, Gossling E, Reuter I, Cheremushkin E, Kel-Margoulis OV, Wingender E (2003). MATCH: a tool for searching transcription factor binding sites in DNA sequences. Nucleic Acids Res.

[19] Schulz E, Gottschling M, Bravo IG, Wittstatt U, Stockfleth E, Nindl I (2009). Genomic characterization of the first insectivoran papillomavirus reveals an unusually long, second non-coding region and indicates a close relationship to Betapapillomavirus. J Gen Virol.

[20] Katoh K, Standley DM (2013). MAFFT multiple sequence alignment software version 7: improvements in performance and usability. Mol Biol Evol.

[21] Suyama M, Torrents D, Bork P (2006). PAL2NAL: robust conversion of protein sequence alignments into the corresponding codon alignments. Nucleic Acids Res.

[22] Castresana J (2000). Selection of conserved blocks from multiple alignments for their use in phylogenetic analysis. Mol Biol Evol.

[23] Rector A, Stevens H, Lacave G, Lemey P, Mostmans S, Salbany A (2008). Genomic characterization of novel dolphin papillomaviruses provides indications for recombination within the Papillomaviridae. Virology.

[24] Gottschling M, Bravo IG, Schulz E, Bracho MA, Deaville R, Jepson PD (2011). Modular organizations of novel cetacean papillomaviruses. Mol Phylogenet Evol.

[25] Robles-Sikisaka R, Rivera R, Nollens HH, St Leger J, Durden WN, Stolen M (2012). Evidence of recombination and positive selection in cetacean papillomaviruses. Virology.

[26] Stamatakis A (2014). RAxML version 8: a tool for phylogenetic analysis and post-analysis of large phylogenies. Bioinformatics.

[27] Huson DH, Bryant D (2006). Application of phylogenetic networks in evolutionary studies. Mol Biol Evol.

[28] Glaunsinger BA, Lee SS, Thomas M, Banks L, Javier R (2000). Interactions of the PDZ-protein MAGI-1 with adenovirus E4-ORF1 and high-risk papillomavirus E6 oncoproteins. Oncogene.

[29] Thomas M, Laura R, Hepner K, Guccione E, Sawyers C, Lasky L (2002). Oncogenic human papillomavirus E6 proteins target the MAGI-2 and MAGI-3 proteins for degradation. Oncogene.

[30] Lee SS, Glaunsinger B, Mantovani F, Banks L, Javier RT (2000). Multi-PDZ domain protein MUPP1 is a cellular target for both adenovirus E4-ORF1 and high-risk papillomavirus type 18 E6 oncoproteins. J Virol.

[31] Nakagawa S, Huibregtse JM (2000). Human scribble (Vartul) is targeted for ubiquitin-mediated degradation by the high-risk papillomavirus E6 proteins and the E6AP ubiquitin-protein ligase. Mol Cell Biol.

[32] Gardiol D, Kuhne C, Glaunsinger B, Lee SS, Javier R, Banks L (1999). Oncogenic human papillomavirus E6 proteins target the discs large tumour suppressor for proteasome-mediated degradation. Oncogene.

[33] Huibregtse JM, Scheffner M, Howley PM (1991). A cellular protein mediates association of p53 with the E6 oncoprotein of human papillomavirus types 16 or 18. EMBO J.

[34] Scheffner M, Huibregtse JM, Vierstra RD, Howley PM (1993). The HPV-16 E6 and E6-AP complex functions as a ubiquitin-protein ligase in the ubiquitination of p53. Cell.

[35] Jing M, Bohl J, Brimer N, Kinter M, Vande Pol SB (2007). Degradation of tyrosine phosphatase PTPN3 (PTPH1) by association with oncogenic human papillomavirus E6 proteins. J Virol.

[36] Spanos WC, Hoover A, Harris GF, Wu S, Strand GL, Anderson ME (2008). The PDZ binding motif of human papillomavirus type 16 E6 induces PTPN13 loss, which allows anchorage-independent growth and synergizes with ras for invasive growth. J Virol.

[37] White EA, Howley PM (2013). Proteomic approaches to the study of papillomavirus-host interactions. Virology.

[38] Kiyono T, Foster SA, Koop JI, McDougall JK, Galloway DA, Klingelhutz AJ (1998). Both Rb/p16INK4a inactivation and telomerase activity are required to immortalize human epithelial cells. Nature.

[39] Lorenz LD, Rivera Cardona J, Lambert PF (2013). Inactivation of p53 rescues the maintenance of high risk HPV DNA genomes deficient in expression of E6. PLoS Pathog.

[40] Dyson N, Howley PM, Munger K, Harlow E (1989). The human papilloma virus-16 E7 oncoprotein is able to bind to the retinoblastoma gene product. Science.

[41] Munger K, Werness BA, Dyson N, Phelps WC, Harlow E, Howley PM (1989). Complex formation of human papillomavirus E7 proteins with the retinoblastoma tumor suppressor gene product. EMBO J.

[42] Saveria Campo M, Moar MH, Jarrett WF, Laird HM (1980). A new papillomavirus associated with alimentary cancer in cattle. Nature.

[43] Jarrett WF, Campo MS, O'Neil BW, Laird HM, Coggins LW (1984). A novel bovine papillomavirus (BPV-6) causing true epithelial papillomas of the mammary gland skin: a member of a proposed new BPV subgroup. Virology.

[44] Hatama S, Ishihara R, Ueda Y, Kanno T, Uchida I (2011). Detection of a novel bovine papillomavirus type 11 (BPV-11) using xipapillomavirus consensus polymerase chain reaction primers. Arch Virol.

[45] Hatama S, Nobumoto K, Kanno T (2008). Genomic and phylogenetic analysis of two novel bovine papillomaviruses, BPV-9 and BPV-10. J Gen Virol.

[46] da Silva FR, Cibulski SP, Daudt C, Weber MN, Guimaraes LL, Streck AF (2016). Novel Bovine papillomavirus type discovered by rolling-circle amplification coupled with next-generation sequencing. PLoS ONE.

[47] Daudt C, da Silva FRC, Cibulski SP, Streck AF, Laurie RE, Munday JS (2019). Bovine papillomavirus 24: a novel member of the genus Xipapillomavirus detected in the Amazon region. Arch Virol.

