# Supplementary material for: Genomic and phylogenetic characterization of ChPV2, a novel goat PV closely related to the Xi-PV1 species infecting bovines
Source: Virol J. 2020 Oct 30;17:167. doi: 10.1186/s12985-020-01440-9 (PMC7602357; doi:10.1186/s12985-020-01440-9)
Supplement: Supplementary file 1 — Additional file 1: Figure S1. Genome organization of ChPV2; Upstream regulatory region, yellow; early genes, green late genes, blue, potential E4 gene and potential E1^E4 splicing patterns, ochre. Figure S2. Presence and position of putative regulatory elements within URR and early gene region of ChPV1 and ChPV2, E1- binding sites (E1BS) are marked by yellow boxes, E2- binding sites (E2BS) perfectly matching the E2BS consensus sequence are marked by red boxes, E2BS with 1 nucleotide mismatch to the E2BS consensus sequence are marked in orange. nucleotide positions of early genes and regulatory elements are depicted below or above the respective element. Figure S3. Maximum likelihood phylogenetic trees of the concatenated E1E2 and concatenated L2L1 nucleotide alignments of 377 PVs. Clade color codes highlight the different PV groups and recombinant taxa: red, Alpha-OmikronPVs; green, Beta-XiPVs; yellow, Lambda-MuPVs; blue, Delta-ZetaPVs; gray, a yet unclassified clade consisting of PVs infecting birds and turtles; aqua-blue, a yet unclassified clade consisting of PVs infecting Caniformia; orange, known recombinant PVs infecting Cetaceans; and pink, possible recombinant PVs. The position of ChPV2 is indicated with a red arrow. Values on branches correspond to ML bootstrap support values. Figure S4. Maximum likelihood phylogenetic trees of the concatenated E1E2 and concatenated L2L1 nucleotide alignments of 324 PVs. Clade color codes highlight the different PV groups: red, Alpha-OmikronPVs; green, Beta-XiPVs; yellow, Lambda-MuPVs; blue, Delta-ZetaPVs; and gray, a yet unclassified clade consisting of PVs infecting birds and turtles. The position of ChPV2 is indicated with a red arrow. Values on branches correspond to ML bootstrap support values. Figure S5. Maximum likelihood phylogenetic trees of the concatenated E1E2 and concatenated L2L1 amino acid alignments of 324 PVs. Clade color codes highlight the different PV groups: red, Alpha-OmikronPVs; green, Beta-XiPVs; yel [file 12985_2020_1440_MOESM1_ESM.pdf]

Supporting data

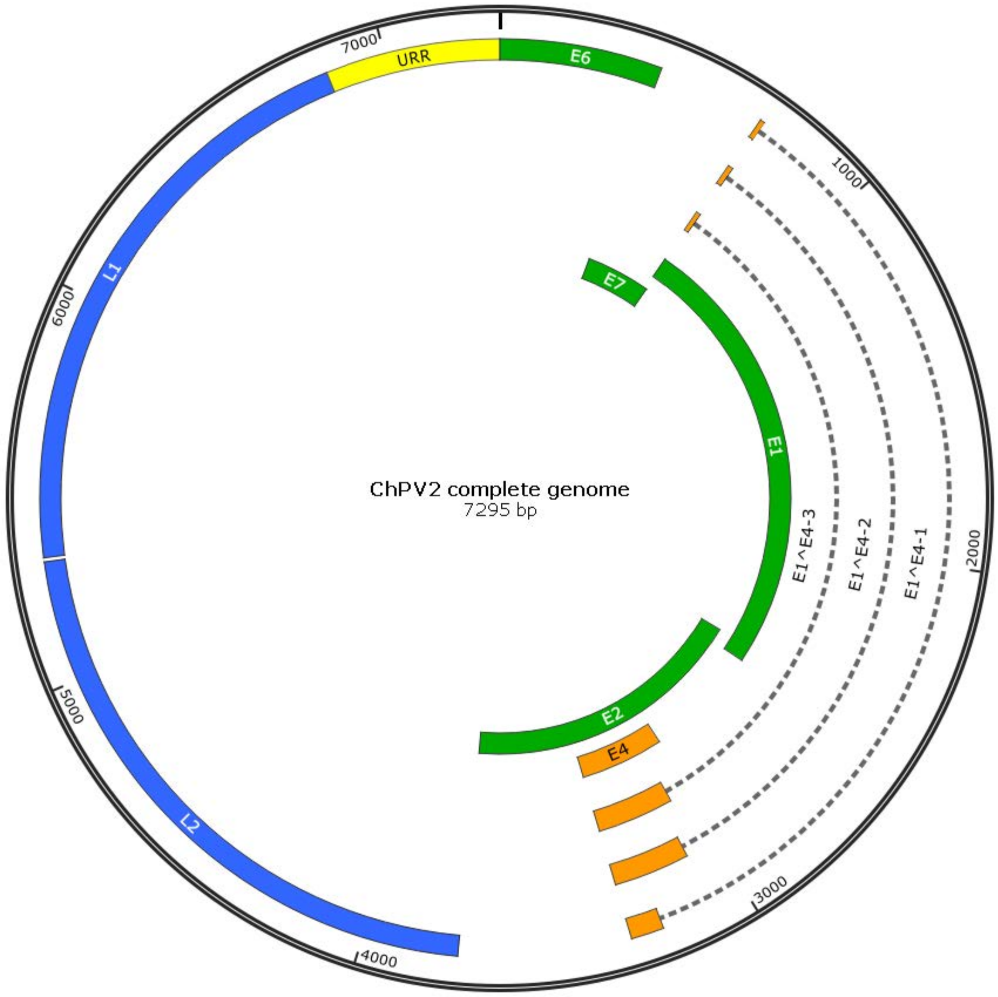

Figure S1: Genome organization of ChPV2, Upstream regulatory region, yellow; early genes, green late genes, blue, potential E4 gene and potential E1^E4 splicing patterns, ochre

Table S1: Genome organization of ChPV2 presenting orf positions, orf length, GC content and amino Acid content of encoded potential proteins and their molecular weight

orf	nt positions	bp	GC content	aa	protein molecular weight kDa
**E6**	1..417	417	41%	138	16.02
**E7**	414..710	297	51%	98	10.81
**E1^E4-1**	700.. 712 and 3052..3311	237	59%	78	10.24
**E1^E4-2**	700.. 712 and 3088..3311	273	58%	90	8.98
**E1^E4-3**	700.. 712 and 3088..3311	99	59%	32	8.12
**E4**	2976..3311	336	55%	111	12.69
**E1**	700..2508	1809	41%	602	68.53
**E2**	2450..3742	1293	48%	430	48.62
**L2**	3755..5311	1557	44%	518	56.74
**L1**	5322..6848	1527	42%	508	57.48
**URR**	6849..7295	447	39%	n.a.	n.a.

Table S2: Nucleotide (nt) identity of ChPV2 genes with the respective genes of closely related PV types

% nt identity	BPV3 XiPV	BPV4 XiPV	BPV6 XiPV	BPV9 XiPV	BPV10 XiPV	BPV11 XiPV	BPV12 XiPV	BPV15 XiPV	BPV17 XiPV	BPV20 XiPV	BPV23 XiPV	BPV24 XiPV	CePV2 XiPV	RtPV2 XiPV	ChPV1 PhiPV	RtiPV1 ?
**E6**	n.a.	n.a.	n.a.	n.a.	n.a.	n.a.	n.a.	n.a.	45.4	62.1	n.a.	n.a.	52.0	62.8	45.4	38.3
**E7**	61.5	60.2	60.6	55.2	65.3	60.2	53.4	58.3	47.8	56.4	59.5	58.1	45.5	56.4	43.8	39.9
**E1**	73.0	71.5	72.1	73.1	70.6	71.7	66.0	70.4	59.5	66.3	73.3	69.7	64.6	68.4	51.6	55.7
**E2**	59.4	59.1	58.4	59.2	62.5	58.5	48.4	59.4	49.5	54.7	58.9	58.5	53.9	55.1	36.2	36.2
**L2**	65.3	64.8	64.2	65.6	66.1	66.9	60.2	64.8	49.3	57.2	63.7	66.1	56.9	58.0	44.3	47.4
**L1**	69.7	70.1	72.0	70.2	71.3	70.3	66.5	67.5	58.3	64.7	69.2	69.6	63.8	67.5	55.3	59.9

Table S3: Amino acid (aa) identity of ChPV2 genes with the respective genes of closely related PV types

% aa identity	BPV3 XiPV	BPV4 XiPV	BPV6 XiPV	BPV9 XiPV	BPV10 XiPV	BPV11 XiPV	BPV12 XiPV	BPV15 XiPV	BPV17 XiPV	BPV20 XiPV	BPV23 XiPV	BPV24 XiPV	CePV2 XiPV	RtPV2 XiPV	ChPV1 PhiPV	RtiPV1 ?
**E6**	n.a.	n.a.	n.a.	n.a.	n.a.	n.a.	n.a.	n.a.	36.9	57.5	n.a.	n.a.	45.8	53.9	30.7	28.9
**E7**	50.0	47.9	52.5	48.4	56.1	48.9	45.9	49.0	38.6	46.9	47.9	44.8	38.6	43.8	39.7	31.6
**E1**	72.6	70.7	72.7	71.8	72.9	69.8	60.0	70.3	52.9	63.9	71.2	69.7	59.1	65.7	44.1	47.9
**E2**	55.4	54.4	52.8	55.0	59.7	53.3	40.7	53.5	41.0	46.4	54.5	52.9	43.2	47.4	28.3	27.8
**L2**	66.4	64.9	63.4	65.9	70.3	67.9	60.4	66.0	47.2	59.3	65.3	65.8	57.3	58.9	40.1	42.5
**L1**	72.9	70.3	75.2	75.0	74.3	71.4	68.6	72.0	53.2	65.6	74.4	69.2	64.0	68.4	52.5	54.6

Table S4: Potential motifs and domains identified in amino acid sequences of ChPV2 proteins

protein	domain/motif/site	consensus sequence	aa position
**E6**	zinc binding motif	C-X_2-C-X_{28}-C-X_2-C	28-64
**E6**	zinc binding motif	C-X_2-C-X_{28}-C-X_2-C	99-135
**E7**	pRB binding motif	LXCXE	24-28
**E7**	CkII phosphorylation site	TLPD	7-10
**E7**	zinc binding motif	C-X_2-C-X_{28}-C-X_2-C	47-83
**E1**	SF3 helicase 1 domain	n.a.	404-554
**E2**	DNA binding helix	GCANTLKCFRRRTSHSHPHK	356-374
**L2**	nuclear localization signal	RKFKRKTK	509-16
**L1**	nuclear localization signal	RAPKRKRK	499-506

Table S5: Potential regulatory elements identified throughout the ChPV2 genome, elements marked with a * have a mismatch to the published consensus sequence for the respective element

element	sequence	nt position
**E2 binding site**	ACC-N_6-GGT	7164.. 7175
**E1 binding site**	GTAGTTGTTGTTAACAACAAT	7201.. 7218
**E2 binding site**	ACC-N_6-GGT	7242.. 7253
**E2 binding site**	ACC-N_6-GGT	7258.. 7269
**TATA Box early promotor**	TATAAA	7273.. 7277
**E2 binding site ***	ACT-N_6-GGT	455.. 466
**E2 binding site ***	ACC-N_6-GTG	481..492
**TATA Box late promotor**	TATAA	555-559
**early pA**	AATAAA	749.. 3754
**E2 binding site**	ACC-N_6-GGT	6789.. 6796
**late pA**	AATAAA	6920.. 6925

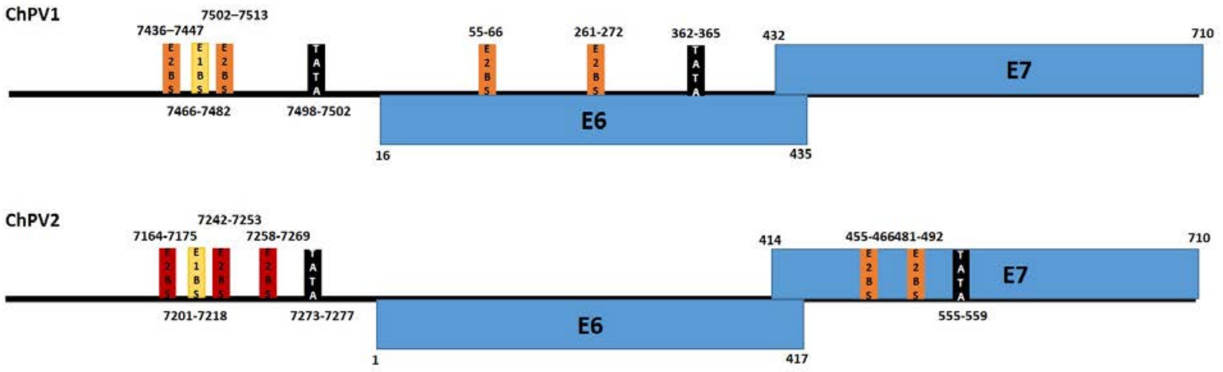

Figure S2: presence and position of regulatory elements within URR and early gene region of ChPV1 and ChPV2, E1- binding sites (E1BS) are marked by yellow boxes, E2- binding sites (E2BS) perfectly matching the E2BS consensus sequence are marked by red boxes, E2BS with 1 nucleotide mismatch to the E2BS consensus sequence are marked in orange. nucleotide positions of early genes and regulatory elements are depicted below or above the respective element.

Table S6: List of PV genomes collected from PaVE (pave.niaid.nih.gov) plus ChPV2

PV_abbreviation	PV_full_name	PV species	PaVE_ID
AaPV1	Alces alces Papillomavirus 1	Delta_1	AaPV1REF
AgPV1	Alouatta guariba Papillomavirus 1	Dyoomikron	AgPV1REF
AmPV1	Ailuropoda melanoleuca Papillomavirus 1	Omega	AmPV1REF
AmPV2	Ailuropoda melanoleuca Papillomavirus 2	Omega	AmPV2REF
AmPV3	Ailuropoda Melanoleuca Papillomavirus 3	Dyonu	AmPV3REF
AmPV4	Ailuropoda melanoleuca Papillomavirus 4	Lambda	AmPV4REF
AsPV1	Apodemus sylvaticus Papillomavirus 1	Pi_2	AsPV1REF
BgPV1	Bos grunniens papillomavirus 1	Delta_4	BgPV1REF
BpPV1	Bettongia penicillata papillomavirus 1	Dyolambda_1	BpPV1REF
BPV1	Bos taurus Papillomavirus 1	Delta_4	BPV1REF
BPV10	Bos taurus Papillomavirus 10	Xi_1	BPV10REF
BPV11	Bos taurus Papillomavirus 11	Xi_1	BPV11REF
BPV12	Bos taurus Papillomavirus 12	Xi_2	BPV12REF
BPV13	Bos taurus Papillomavirus 13	Delta_4	BPV13REF
BPV14	Bovine papillomavirus 14	Delta_4	BPV14REF
BPV15	Bovine papillomavirus 15	Xi_1	BPV15REF
BPV16	Bovine Papillomavirus 16	Dyokappa	BPV16REF
BPV17	Bovine Papillomavirus 17	Xi	BPV17REF
BPV18	Bovine Papillomavirus 18	Dyokappa	BPV18REF
BPV19	Bovine Papillomavirus 19	Unclassified	BPV19REF
BPV2	Bos taurus Papillomavirus 2	Delta_4	BPV2REF
BPV20	Bovine Papillomavirus 20	Xi	BPV20REF
BPV21	Bovine Papillomavirus 21	Unclassified	BPV21REF
BPV22	Bos taurus Papillomavirus 22	Dyokappa	BPV22REF
BPV23	Bos taurus Papillomavirus 23	Xi	BPV23REF
BPV24	Bos Taurus Papillomavirus 24	Xi	BPV24REF
BPV3	Bos taurus Papillomavirus 3	Xi_1	BPV3REF
BPV4	Bos taurus Papillomavirus 4	Xi_1	BPV4REF
BPV5	Bos taurus Papillomavirus 5	Epsilon_1	BPV5REF
BPV6	Bos taurus Papillomavirus 6	Xi_1	BPV6REF
BPV7	Bos taurus Papillomavirus 7	Dyoxi_1	BPV7REF
BPV8	Bos taurus Papillomavirus 8	Epsilon_1	BPV8REF
BPV9	Bos taurus Papillomavirus 9	Xi_1	BPV9REF
CcanPV1	Castor canadensis papillomavirus 1	Dyosigma_1	CcanPV1REF
CcaPV1	Capreolus capreolus Papillomavirus 1	Delta_5	CcaPV1REF
CcPV1	Caretta caretta Papillomavirus 1	Dyozeta_1	CcPV1REF
CcrPV1	Crocuta crocuta papillomavirus 1	Lambda_5	CcrPV1REF
CdPV1	Camelus dromedarius papillomavirus 1	Delta_6	CdPV1REF
CdPV2	Camelus dromedarius papillomavirus 2	Delta_6	CdPV2REF
CePV1	Cervus elaphus Papillomavirus 2	Epsilon	CePV1REF
CePV2	Cervus elaphus Papillomavirus 1	Xi	CePV2REF
CgPV1	Colobus guereza Papillomavirus 1	Alpha_14	CgPV1REF
CgPV2	Colobus guereza Papillomavirus 2	Beta_1	CgPV2REF
ChPV1	Capra hircus Papillomavirus 1	Phi_1	ChPV1REF
ChPV2	Capra hircus Papillomavirus 2	Unclassified	ChPV2REF

CmPV1	Chelonia mydas Papillomavirus 1	Dyozeta_1	CmPV1REF
CPV1	Canis familiaris oral Papillomavirus	Lambda_2	CPV1REF
CPV10	Canis familiaris papillomavirus 10	Chi_3	CPV10REF
CPV11	Canis familiaris papillomavirus 11	Chi_1	CPV11REF
CPV12	Canis familiaris papillomavirus 12	Chi_1	CPV12REF
CPV13	Canis familiaris papillomavirus 13	Tau_2	CPV13REF
CPV14	Canis familiaris papillomavirus 14	Chi_3	CPV14REF
CPV15	Canis familiaris papillomavirus 15	Chi_3	CPV15REF
CPV16	Canine papillomavirus 16	Chi_2	CPV16REF
CPV17	Canine Papillomavirus 17	Tau	CPV17REF
CPV18	Canine Papillomavirus 18	Chi	CPV18REF
CPV19	Canine Papillomavirus 19	Tau	CPV19REF
CPV2	Canis familiaris Papillomavirus 2	Tau_1	CPV2REF
CPV20	Canine Papillomavirus 20	Chi	CPV20REF
CPV3	Canis familiaris Papillomavirus 3	Chi_1	CPV3REF
CPV4	Canis familiaris Papillomavirus 4	Chi_2	CPV4REF
CPV5	Canis familiaris Papillomavirus 5	Chi_1	CPV5REF
CPV6	Canis familiaris Papillomavirus 6	Lambda_3	CPV6REF
CPV7	Canis familiaris Papillomavirus 7	Tau_1	CPV7REF
CPV8	Canis familiaris papillomavirus 8	Chi_3	CPV8REF
CPV9	Canis familiaris papillomavirus 9	Chi_1	CPV9REF
DdPV1	Delphinus delphis papillomavirus	Upsilon_1	DdPV1REF
EaPV1	Equus asinus papillomavirus	Dyochi_1	EaPV1REF
EcPV1	Equus caballus Papillomavirus 1	Zeta_1	EcPV1REF
EcPV2	Equus caballus Papillomavirus 2	Dyoiota_1	EcPV2REF
EcPV3	Equus caballus papillomavirus 3	Dyorho_1	EcPV3REF
EcPV4	Equus caballus papillomavirus 4	Dyoiota_2	EcPV4REF
EcPV5	Equus caballus papillomavirus 5	Dyoiota_2	EcPV5REF
EcPV6	Equus caballus papillomavirus 6	Dyorho_1	EcPV6REF
EcPV7	Equus caballus papillomavirus 7	Dyorho_1	EcPV7REF
EcPV8	Equus caballus Papillomavirus 8	Unclassified	EcPV8REF
EdPV1	Erethizon dorsatum Papillomavirus 1	Sigma_1	EdPV1REF
EePV1	Erinaceus europaeus Papillomavirus 1	Dyoeta_1	EePV1REF
EhPV1	Eidolon helvum papillomavirus 1	Dyoupsilon_1	EhPV1REF
EhPV2	Eidolon helvum Papillomavirus 2	Psi	EhPV2REF
EhPV3	Eidolon helvum Papillomavirus 3	Psi	EhPV3REF
ElPV1	Enhydra lutris papillomavirus 1	Lambda_4	ElPV1REF
EsPV1	Eptesicus serotinus papillomavirus 1	Dyopsi_1	EsPV1REF
EsPV2	Eptesicus serotinus papillomavirus 2	Dyoomega_1	EsPV2REF
EsPV3	Eptesicus serotinus papillomavirus 3	Dyopsi_1	EsPV3REF
FcaPV1	Felis domesticus Papillomavirus 1	Lambda_1	FcaPV1REF
FcaPV2	Felis domesticus Papillomavirus 2	Dyotheta_1	FcaPV2REF
FcaPV3	Felis catus papillomavirus 3	Tau_3	FcaPV3REF
FcaPV4	Felis catus papillomavirus 4	Tau_3	FcaPV4REF
FcaPV5	Felis catus Papillomavirus 5	Tau	FcaPV5REF
FcPV1	Fringilla coelebs Papillomavirus	Eta_1	FcPV1REF
FgPV1	Fulmarus glacialis papillomavirus 1	Treiszeta	FgPV1REF
FlPV1	Francolinus leucoscepus Papillomavirus 1	Dyoepsilon_1	FlPV1REF

GcPV1	Giraffa camelopardalis Papillomavirus 1	Delta	GcPV1REF
HPV1	Human papillomavirus 1	Mu_1	HPV1REF
HPV10	Human papillomavirus 10	Alpha_2	HPV10REF
HPV100	Human papillomavirus 100	Beta_2	HPV100REF
HPV101	Human papillomavirus 101	Gamma_6	HPV101REF
HPV102	Human papillomavirus 102	Alpha_3	HPV102REF
HPV103	Human papillomavirus 103	Gamma_6	HPV103REF
HPV104	Human papillomavirus 104	Beta_2	HPV104REF
HPV105	Human papillomavirus 105	Beta_1	HPV105REF
HPV106	Human papillomavirus 106	Alpha_14	HPV106REF
HPV107	Human papillomavirus 107	Beta_2	HPV107REF
HPV108	Human Papillomavirus 108	Gamma_6	HPV108REF
HPV109	Human papillomavirus 109	Gamma_7	HPV109REF
HPV11	Human papillomavirus 11	Alpha_10	HPV11REF
HPV110	Human papillomavirus 110	Beta_2	HPV110REF
HPV111	Human papillomavirus 111	Beta_2	HPV111REF
HPV112	Human papillomavirus 112	Gamma_8	HPV112REF
HPV113	Human papillomavirus 113	Beta_2	HPV113REF
HPV114	Human papillomavirus 114	Alpha_3	HPV114REF
HPV115	Human papillomavirus 115	Beta_3	HPV115REF
HPV116	Human papillomavirus 116	Gamma_9	HPV116REF
HPV117	Human papillomavirus 117	Alpha_2	HPV117REF
HPV118	Human papillomavirus 118	Beta_1	HPV118REF
HPV119	Human papillomavirus 119	Gamma_8	HPV119REF
HPV12	Human papillomavirus 12	Beta_1	HPV12REF
HPV120	Human papillomavirus 120	Beta_2	HPV120REF
HPV121	Human papillomavirus 121	Gamma_10	HPV121REF
HPV122	Human papillomavirus 122	Beta_2	HPV122REF
HPV123	Human papillomavirus 123	Gamma_7	HPV123REF
HPV124	Human papillomavirus 124	Beta_1	HPV124REF
HPV125	Human papillomavirus 125	Alpha_2	HPV125REF
HPV126	Human papillomavirus 126	Gamma_11	HPV126REF
HPV127	Human papillomavirus 127	Gamma_12	HPV127REF
HPV128	Human papillomavirus 128	Gamma_13	HPV128REF
HPV129	Human papillomavirus 129	Gamma_9	HPV129REF
HPV13	Human papillomavirus 13	Alpha_10	HPV13REF
HPV130	Human papillomavirus 130	Gamma_10	HPV130REF
HPV131	Human papillomavirus 131	Gamma_14	HPV131REF
HPV132	Human papillomavirus 132	Gamma_12	HPV132REF
HPV133	Human papillomavirus 133	Gamma_10	HPV133REF
HPV134	Human papillomavirus 134	Gamma_7	HPV134REF
HPV135	Human papillomavirus 135	Gamma_15	HPV135REF
HPV136	Human papillomavirus 136	Gamma_11	HPV136REF
HPV137	Human papillomavirus 137	Gamma_16	HPV137REF
HPV138	Human papillomavirus 138	Gamma_7	HPV138REF
HPV139	Human papillomavirus 139	Gamma_7	HPV139REF
HPV14	Human papillomavirus 14	Beta_1	HPV14REF
HPV140	Human papillomavirus 140	Gamma_11	HPV140REF

HPV141	Human papillomavirus 141	Gamma_11	HPV141REF
HPV142	Human papillomavirus 142	Gamma_10	HPV142REF
HPV143	Human papillomavirus 143	Beta_1	HPV143REF
HPV144	Human papillomavirus 144	Gamma_17	HPV144REF
HPV145	Human papillomavirus 145	Beta_2	HPV145REF
HPV146	Human papillomavirus 146	Gamma_15	HPV146REF
HPV147	Human papillomavirus 147	Gamma_8	HPV147REF
HPV148	Human papillomavirus 148	Gamma_12	HPV148REF
HPV149	Human papillomavirus 149	Gamma_7	HPV149REF
HPV15	Human papillomavirus 15	Beta_2	HPV15REF
HPV150	Human papillomavirus 150	Beta_5	HPV150REF
HPV151	Human papillomavirus 151	Beta_2	HPV151REF
HPV152	Human papillomavirus 152	Beta_1	HPV152REF
HPV153	Human papillomavirus 153	Gamma_13	HPV153REF
HPV154	Human papillomavirus 154	Gamma_11	HPV154REF
HPV155	Human papillomavirus 155	Gamma_7	HPV155REF
HPV156	Human papillomavirus 156	Gamma_18	HPV156REF
HPV157	Human Papillomavirus 157	Gamma	HPV157REF
HPV158	Human Papillomavirus 158	Gamma	HPV158REF
HPV159	Human papillomavirus 159	Beta_2	HPV159REF
HPV16	Human papillomavirus 16	Alpha_9	HPV16REF
HPV160	Human papillomavirus 160	Alpha_2	HPV160REF
HPV161	Human papillomavirus 161	Gamma_19	HPV161REF
HPV162	Human papillomavirus 162	Gamma_19	HPV162REF
HPV163	Human papillomavirus 163	Gamma_20	HPV163REF
HPV164	Human papillomavirus 164	Gamma_8	HPV164REF
HPV165	Human Papillomavirus 165	Gamma_12	HPV165REF
HPV166	Human papillomavirus 166	Gamma_19	HPV166REF
HPV167	Human papillomavirus 167	Gamma_21	HPV167REF
HPV168	Human papillomavirus 168	Gamma_8	HPV168REF
HPV169	Human papillomavirus 169	Gamma_11	HPV169REF
HPV17	Human papillomavirus 17	Beta_2	HPV17REF
HPV170	Human papillomavirus 170	Gamma_7	HPV170REF
HPV171	Human papillomavirus 171	Gamma_11	HPV171REF
HPV172	Human papillomavirus 172	Gamma_22	HPV172REF
HPV173	Human papillomavirus 173	Gamma_1	HPV173REF
HPV174	Human papillomavirus 174	Beta_2	HPV174REF
HPV175	Human papillomavirus 175	Gamma_23	HPV175REF
HPV178	Human Papillomavirus 178	Gamma_24	HPV178REF
HPV179	Human papillomavirus type 179	Gamma_15	HPV179REF
HPV18	Human papillomavirus 18	Alpha_7	HPV18REF
HPV180	Human papillomavirus 180	Gamma_10	HPV180REF
HPV184	Human papillomavirus type 184	Gamma_25	HPV184REF
HPV187	Human Papillomavirus 187	Gamma_26	HPV187REF
HPV19	Human papillomavirus 19	Beta_1	HPV19REF
HPV197	Human papillomavirus type 197	Gamma_24	HPV197REF
HPV199	Human papillomavirus type 199	Gamma_12	HPV199REF
HPV2	Human papillomavirus 2	Alpha_4	HPV2REF

HPV20	Human papillomavirus 20	Beta_1	HPV20REF
HPV200	Human papillomavirus 200	Gamma_2	HPV200REF
HPV201	Human papillomavirus 201	Gamma_27	HPV201REF
HPV202	Human papillomavirus 202	Gamma_11	HPV202REF
HPV203	Human Papillomavirus 203	Gamma	HPV203REF
HPV204	Human papillomavirus 204	Mu_3	HPV204REF
HPV205	Human Papillomavirus 205	Gamma	HPV205REF
HPV209	Human Papillomavirus 209	Beta	HPV209REF
HPV21	Human papillomavirus 21	Beta_1	HPV21REF
HPV211	Human Papillomavirus mCT02_8	Gamma	HPV211REF
HPV212	Human Papillomavirus mCT023_1	Gamma	HPV212REF
HPV213	Human Papillomavirus 213	Gamma	HPV213REF
HPV214	Human Papillomavirus 214	Gamma	HPV214REF
HPV215	Human Papillomavirus 215	Gamma	HPV215REF
HPV216	Human Papillomavirus 216	Gamma	HPV216REF
HPV22	Human papillomavirus 22	Beta_2	HPV22REF
HPV223	Human Papillomavirus 223	Gamma	HPV223REF
HPV225	Human Papillomavirus 225	Gamma	HPV225REF
HPV23	Human papillomavirus 23	Beta_2	HPV23REF
HPV24	Human papillomavirus 24	Beta_1	HPV24REF
HPV25	Human papillomavirus 25	Beta_1	HPV25REF
HPV26	Human papillomavirus 26	Alpha_5	HPV26REF
HPV27	Human papillomavirus 27	Alpha_4	HPV27REF
HPV28	Human papillomavirus 28	Alpha_2	HPV28REF
HPV29	Human papillomavirus 29	Alpha_2	HPV29REF
HPV3	Human papillomavirus 3	Alpha_2	HPV3REF
HPV30	Human papillomavirus 30	Alpha_6	HPV30REF
HPV31	Human papillomavirus 31	Alpha_9	HPV31REF
HPV32	Human papillomavirus 32	Alpha_1	HPV32REF
HPV33	Human papillomavirus 33	Alpha_9	HPV33REF
HPV34	Human papillomavirus 34	Alpha_11	HPV34REF
HPV35	Human papillomavirus 35	Alpha_9	HPV35REF
HPV36	Human papillomavirus 36	Beta_1	HPV36REF
HPV37	Human papillomavirus 37	Beta_2	HPV37REF
HPV38	Human papillomavirus 38	Beta_2	HPV38REF
HPV39	Human papillomavirus 39	Alpha_7	HPV39REF
HPV4	Human papillomavirus 4	Gamma_1	HPV4REF
HPV40	Human papillomavirus 40	Alpha_8	HPV40REF
HPV41	Human papillomavirus 41	Nu_1	HPV41REF
HPV42	Human papillomavirus 42	Alpha_1	HPV42REF
HPV43	Human papillomavirus 43	Alpha_8	HPV43REF
HPV44	Human papillomavirus 44	Alpha_10	HPV44REF
HPV45	Human papillomavirus 45	Alpha_7	HPV45REF
HPV47	Human papillomavirus 47	Beta_1	HPV47REF
HPV48	Human papillomavirus 48	Gamma_2	HPV48REF
HPV49	Human papillomavirus 49	Beta_3	HPV49REF
HPV5	Human Papillomavirus 5	Beta_1	HPV5REF
HPV50	Human papillomavirus 50	Gamma_3	HPV50REF

HPV51	Human papillomavirus 51	Alpha_5	HPV51REF
HPV52	Human papillomavirus 52	Alpha_9	HPV52REF
HPV53	Human papillomavirus 53	Alpha_6	HPV53REF
HPV54	Human papillomavirus 54	Alpha_13	HPV54REF
HPV56	Human papillomavirus 56	Alpha_6	HPV56REF
HPV57	Human papillomavirus 57	Alpha_4	HPV57REF
HPV58	Human papillomavirus 58	Alpha_9	HPV58REF
HPV59	Human papillomavirus 59	Alpha_7	HPV59REF
HPV6	Human papillomavirus 6	Alpha_10	HPV6REF
HPV60	Human papillomavirus 60	Gamma_4	HPV60REF
HPV61	Human papillomavirus 61	Alpha_3	HPV61REF
HPV62	Human papillomavirus 62	Alpha_3	HPV62REF
HPV63	Human papillomavirus 63	Mu_2	HPV63REF
HPV65	Human papillomavirus 65	Gamma_1	HPV65REF
HPV66	Human papillomavirus 66	Alpha_6	HPV66REF
HPV67	Human papillomavirus 67	Alpha_9	HPV67REF
HPV68	Human papillomavirus 68	Alpha_7	HPV68REF
HPV69	Human papillomavirus 69	Alpha_5	HPV69REF
HPV7	Human papillomavirus 7	Alpha_8	HPV7REF
HPV70	Human papillomavirus 70	Alpha_7	HPV70REF
HPV71	Human papillomavirus 71	Alpha_14	HPV71REF
HPV72	Human papillomavirus 72	Alpha_3	HPV72REF
HPV73	Human papillomavirus 73	Alpha_11	HPV73REF
HPV74	Human papillomavirus 74	Alpha_10	HPV74REF
HPV75	Human papillomavirus 75	Beta_3	HPV75REF
HPV76	Human papillomavirus 76	Beta_3	HPV76REF
HPV77	Human papillomavirus 77	Alpha_2	HPV77REF
HPV78	Human papillomavirus 78	Alpha_2	HPV78REF
HPV8	Human papillomavirus 8	Beta_1	HPV8REF
HPV80	Human papillomavirus 80	Beta_2	HPV80REF
HPV81	Human papillomavirus 81	Alpha_3	HPV81REF
HPV82	Human papillomavirus 82	Alpha_5	HPV82REF
HPV83	Human papillomavirus 83	Alpha_3	HPV83REF
HPV84	Human papillomavirus 84	Alpha_3	HPV84REF
HPV85	Human papillomavirus 85	Alpha_7	HPV85REF
HPV86	Human papillomavirus 86	Alpha_3	HPV86REF
HPV87	Human papillomavirus 87	Alpha_3	HPV87REF
HPV88	Human papillomavirus 88	Gamma_5	HPV88REF
HPV89	Human papillomavirus 89	Alpha_3	HPV89REF
HPV9	Human papillomavirus 9	Beta_2	HPV9REF
HPV90	Human papillomavirus 90	Alpha_14	HPV90REF
HPV91	Human papillomavirus 91	Alpha_8	HPV91REF
HPV92	Human papillomavirus 92	Beta_4	HPV92REF
HPV93	Human papillomavirus 93	Beta_1	HPV93REF
HPV94	Human papillomavirus 94	Alpha_2	HPV94REF
HPV95	Human papillomavirus 95	Gamma_1	HPV95REF
HPV96	Human papillomavirus 96	Beta_5	HPV96REF
HPV97	Human papillomavirus 97	Alpha_7	HPV97REF

HPV98	Human papillomavirus 98	Beta_1	HPV98REF
HPV99	Human papillomavirus 99	Beta_1	HPV99REF
LrPV1	Lynx rufus Papillomavirus 1	Lambda_1	LrPV1REF
LwPV1	Leptonychotes weddellii Papillomavirus 1	Unclassified	LwPV1REF
LwPV2	Leptonychotes weddellii Papillomavirus 2	Unclassified	LwPV2REF
LwPV3	Leptonychotes weddellii Papillomavirus 3	Dyonu	LwPV3REF
LwPV4	Leptonychotes weddellii Papillomavirus 4	Dyotheta	LwPV4REF
LwPV5	Leptonychotes weddellii Papillomavirus 5	Tau	LwPV5REF
LwPV6	Leptonychotes weddellii Papillomavirus 6	Omega	LwPV6REF
LwPV7	Leptonychotes weddellii Papillomavirus 7	Omega	LwPV7REF
MaPV1	Mesocricetus auratus Papillomavirus 1	Pi_2	MaPV1REF
McPV2	Mastomys coucha Papillomavirus 2	Pi_2	McPV2REF
MfPV1	Macaca fascicularis Papillomavirus 1	Beta_1	MfPV1REF
MfPV10	Macaca fascicularis Papillomavirus 10	Alpha_12	MfPV10REF
MfPV11	Macaca fascicularis Papillomavirus 11	Alpha_12	MfPV11REF
MfPV2	Macaca fascicularis Papillomavirus 2	Beta_6	MfPV2REF
MfPV3	Macaca fascicularis Papillomavirus 3	Alpha_12	MfPV3REF
MfPV4	Macaca fascicularis Papillomavirus 4	Alpha_12	MfPV4REF
MfPV5	Macaca fascicularis Papillomavirus 5	Alpha_12	MfPV5REF
MfPV6	Macaca fascicularis Papillomavirus 6	Alpha_12	MfPV6REF
MfPV7	Macaca fascicularis Papillomavirus 7	Alpha_12	MfPV7REF
MfPV8	Macaca fascicularis Papillomavirus 8	Alpha_12	MfPV8REF
MfPV9	Macaca fascicularis Papillomavirus 9	Alpha_12	MfPV9REF
MfuPV1	Macaca fuscata Papillomavirus 1	Alpha	MfuPV1REF
MmiPV1	Micromys minutus Papillomavirus 1	Pi_2	MmiPV1REF
MmoPV1	Molossus molossus Papillomavirus 1	Unclassified	MmoPV1REF
MmPV1	Macaca mulata Papillomavirus 1	Alpha_12	MmPV1REF
MmuPV1	Mus musculus papillomavirus type	Pi_2	MmuPV1REF
MnPV1	Mastomys natalensis Papillomavirus 1	Iota_1	MnPV1REF
MpPV1	Mustela putorius papillomavirus 1	Tau	MpPV1REF
MrPV1	Myotis ricketti papillomavirus 1	Unclassified	MrPV1REF
MscPV1	Miniopterus schreibersii Papillomavirus 1	Unclassified	MscPV1REF
MscPV2	Miniopterus schreibersii papillomavirus 2	Dyotau_1	MscPV2REF
MsPV1	Morelia spilota papillomavirus 1	Dyomu_1	MsPV1REF
OaPV1	Ovis aries Papillomavirus 1	Delta_3	OaPV1REF
OaPV2	Ovis aries Papillomavirus 2	Delta_3	OaPV2REF
OaPV3	Ovis aries Papillomavirus 3	Dyokappa_1	OaPV3REF
OaPV4	Ovis aries Papillomavirus 4	Delta	OaPV4REF
OcPV1	Oryctolagus cuniculus Papillomavirus 2	Kappa_1	OcPV1REF
OvPV1	Odocoileus virginianus Papillomavirus 1	Delta_2	OvPV1REF
PaPV1	Pygoscelis adeliae papillomavirus	Treisepsilon_1	PaPV1REF
PaPV2	Pygoscelis adeliae Papillomavirus 2	Treisepsilon	PaPV2REF
PcPV1	Puma concolor Papillomavirus 1	Lambda_1	PcPV1REF
PePV1	Psittacus erithacus Papillomavirus 1	Theta_1	PePV1REF
PhPV1	Papio hamadryas papillomavirus 1	Alpha_12	PhPV1REF
PlpPV1	Panthera leo persica Papillomavirus	Lambda_1	PlpPV1REF
PlPV1	Procyon lotor Papillomavirus 1	Lambda_4	PlPV1REF
PmPV1	Peromyscus papillomavirus 1	Iota	PmPV1REF

PphPV1	Phocoena phocoena papillomavirus	Omikron_1	PphPV1REF
PphPV2	Phocoena phocoena papillomavirus	Upsilon_3	PphPV2REF
PphPV4	Phocoena phocoena papillomavirus	Dyopi_1	PphPV4REF
PpPV1	Pan paniscus Papillomavirus 1	Alpha_10	PpPV1REF
PpuPV1	Pudu Papillomavirus 1	Unclassified	PpuPV1REF
PsPV1	Phocoena spinipinnis Papillomavirus	Omikron_1	PsPV1REF
PsuPV1	Phodopus sungorus papillomavirus type	Pi_1	PsuPV1REF
RalPV1	Rusa alfredi Papillomavirus 1	Delta	RalPV1REF
RaPV1	Rousettus aegyptiacus Papillomavirus 1	Psi_1	RaPV1REF
RfPV1	Rhinolophus ferrumequinum papillomavirus type	Treisdelta_1	RfPV1REF
RnPV1	Rattus norvegicus Papillomavirus 1	Pi_2	RnPV1REF
RnPV2	Rattus norvegicus Papillomavirus 2	Iota_1	RnPV2REF
RnPV3	Rattus norvegicus Papillomavirus 3	Iota_1	RnPV3REF
RrPV1	Rupicapra rupicapra papillomavirus 1	Dyokappa_2	RrPV1REF
RtiPV1	Rusa timorensis Papillomavirus 1	Unclassified	RtiPV1REF
RtiPV2	Rusa timorensis Papillomavirus 2	Unclassified	RtiPV2REF
RtPV1	Rangifer tarandus Papillomavirus 1	Delta_1	RtPV1REF
RtPV2	Rangifer tarandus papillomavirus 2	Xi_3	RtPV2REF
SaPV1	Sparus aurata Papillomavirus 1	Unclassified	SaPV1REF
ScPV1	Serinus canaria Papillomavirus 1	Eta	ScPV1REF
SfPV1	Sylvilagus floridanus Papillomavirus 1	Kappa_2	SfPV1REF
SscPV1	Saimiri sciureus papillomavirus 1	Dyoomikron_1	SscPV1REF
SscPV2	Saimiri sciureus papillomavirus 2	Dyoomikron_1	SscPV2REF
SscPV3	Saimiri sciureus papillomavirus 3	Dyoomikron_1	SscPV3REF
SsPV1	Sus scrofa Papillomavirus 1	Dyodelta_1	SsPV1REF
SsPV2	Sus scrofa Papillomavirus 2	Unclassified	SsPV2REF
TePV1	Talpa europaea papillomavirus 1	Dyophi_1	TePV1REF
TmPV1	Trichechus manatus latirostris Papillomavirus	Rho_1	TmPV1REF
TmPV2	Trichechus manatus latirostris papillomavirus	Rho_1	TmPV2REF
TmPV3	Trichechus manatus papillomavirus 3	Rho_2	TmPV3REF
TmPV4	Trichechus manatus papillomavirus 4	Rho_2	TmPV4REF
TtPV1	Tursiops truncatus Papillomavirus 1	Upsilon_1	TtPV1REF
TtPV2	Tursiops truncatus Papillomavirus 2	Upsilon_2	TtPV2REF
TtPV3	Tursiops truncatus Papillomavirus 3	Upsilon_1	TtPV3REF
TtPV4	Tursiops truncatus papillomavirus 4	Upsilon_1	TtPV4REF
TtPV5	Tursiops truncatus papillomavirus 5	Omikron_1	TtPV5REF
TtPV6	Tursiops truncatus papillomavirus 6	Omikron_1	TtPV6REF
TtPV7	Tursiops truncatus papillomavirus 7	Upsilon_1	TtPV7REF
TtPV8	Tursiops truncatus Papillomavirus 8	Dyopi	TtPV8REF
TtPV9	Tursiops truncatus Papillomavirus 9	Omikron	TtPV9REF
UmPV1	Ursus maritimus Papillomavirus 1	Omega_1	UmPV1REF
UuPV1	Uncia uncia Papillomavirus 1	Lambda_1	UuPV1REF
VvPV1	Vulpes vulpes papillomavirus 1	Treiseta	VvPV1REF
ZcPV1	Zalophus californianus papillomavirus 1	Dyonu_1	ZcPV1REF

Fig. S2

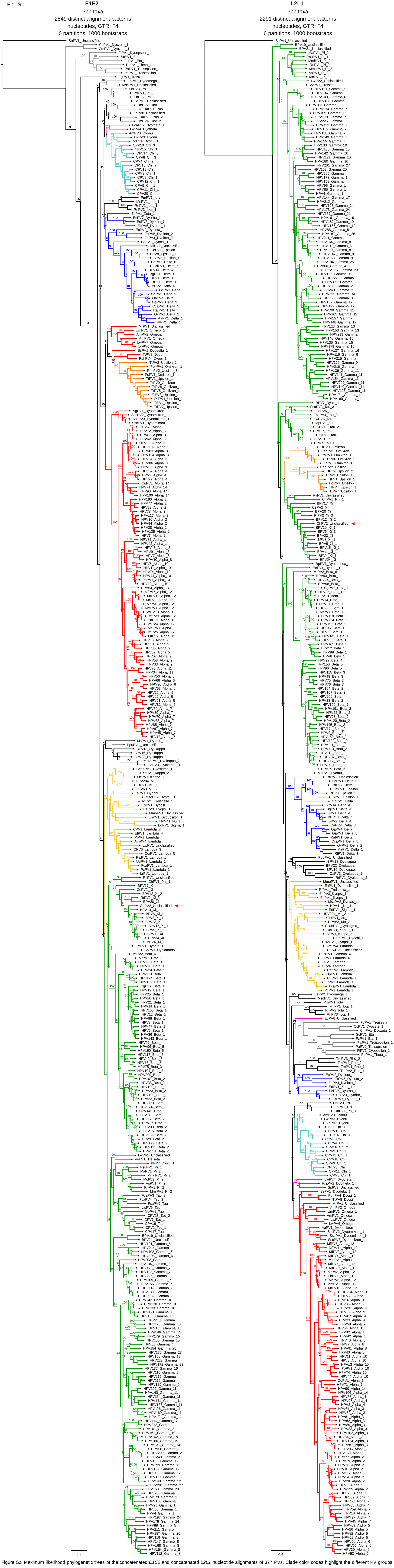

Figure S2. Maximum likelihood phylogenetic trees of the concatenated *E1E2* and concatenated *L2L1* nucleotide alignments of 377 PVs. Clade color codes highlight the different PV groups and recombinant taxa: red, Alpha-OmikronPVs; green, Beta-XiPVs; yellow, Lambda-MuPVs; blue, Delta-ZetaPVs; gray, a yet unclassified clade consisting of PVs infecting birds and turtles; aqua-blue, a yet unclassified clade consisting of PVs infecting Caniformia; orange, known recombinant PVs infecting Cetaceans; and pink, possible recombinant PVs. The position of ChPV2 is indicated with a red arrow. Values on branches correspond to ML bootstrap support values.

Fig. S3

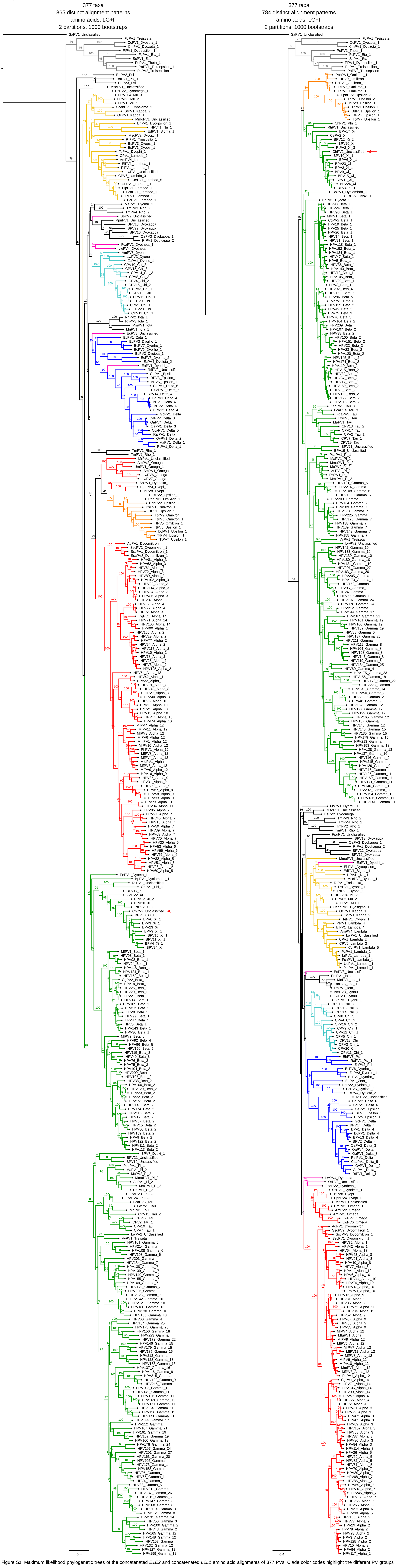

Figure S3. Maximum likelihood phylogenetic trees of the concatenated *E1E2* and concatenated *L2L1* amino acid alignments of 377 PVs. Clade color codes highlight the different PV groups and recombinant taxa: red, Alpha-OmikronPVs; green, Beta-XiPVs; yellow, Lambda-MuPVs; blue, Delta-ZetaPVs; gray, a yet unclassified clade consisting of PVs infecting birds and turtles; aqua-blue, a yet unclassified clade consisting of PVs infecting Caniformia; orange, known recombinant PVs infecting Cetaceans; and pink, possible recombinant PVs. The position of ChPV2 is indicated with a red arrow. Values on branches correspond to ML bootstrap support values.

Fig. S4

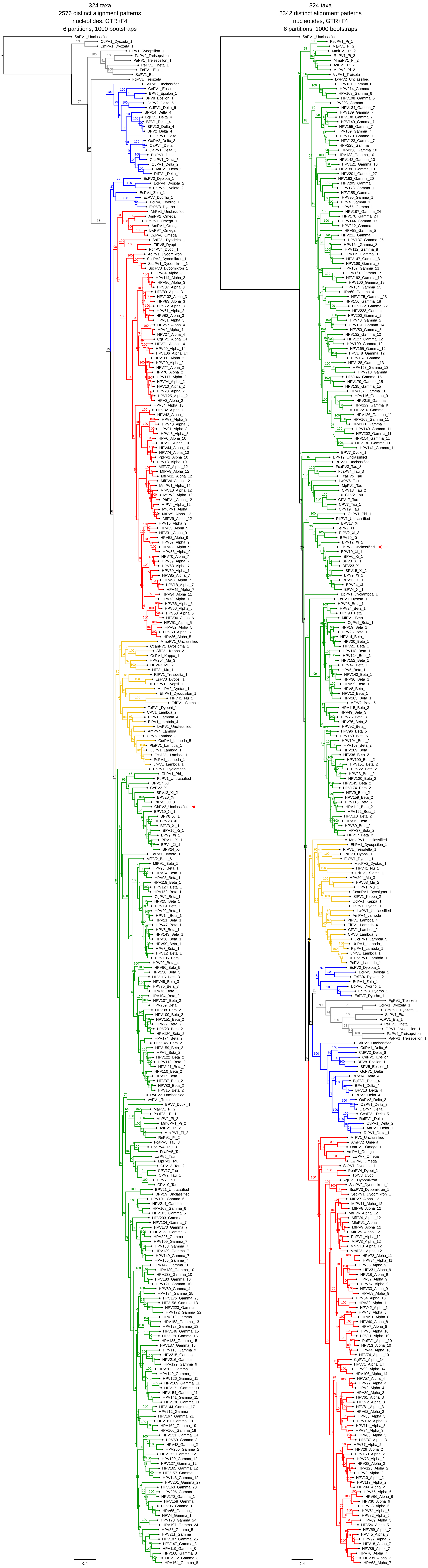

Figure S4. Maximum likelihood phylogenetic trees of the concatenated *E1E2* and concatenated *L2L1* nucleotide alignments of 324 PVs. Clade color codes highlight the different PV groups: red, Alpha-OmikronPVs; green, Beta-XiPVs; yellow, Lambda-MuPVs; blue, Delta-ZetaPVs; and gray, a yet unclassified clade consisting of PVs infecting birds and turtles. The position of ChPV2 is indicated with a red arrow. Values on branches correspond to ML bootstrap support values.

Fig. S5

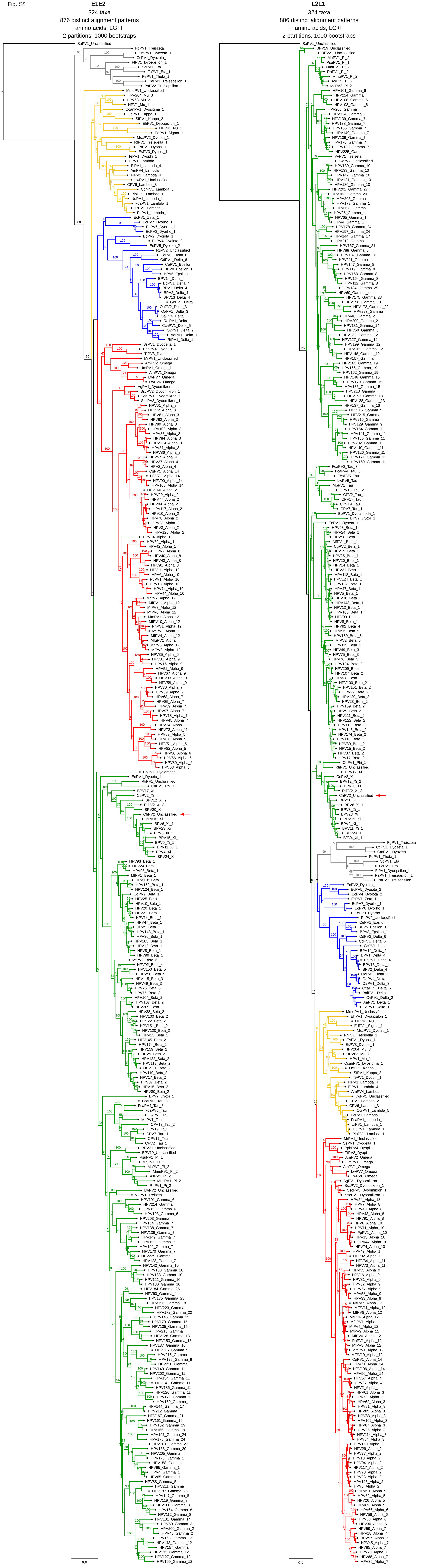

Figure S5. Maximum likelihood phylogenetic trees of the concatenated *E1E2* and concatenated *L2L1* amino acid alignments of 324 PVs. Clade color codes highlight the different PV groups: red, Alpha-OmikronPVs; green, Beta-XiPVs; yellow, Lambda-MuPVs; blue, Delta-ZetaPVs; and gray, a yet unclassified clade consisting of PVs infecting birds and turtles. The position of ChPV2 is indicated with a red arrow. Values on branches correspond to ML bootstrap support values.

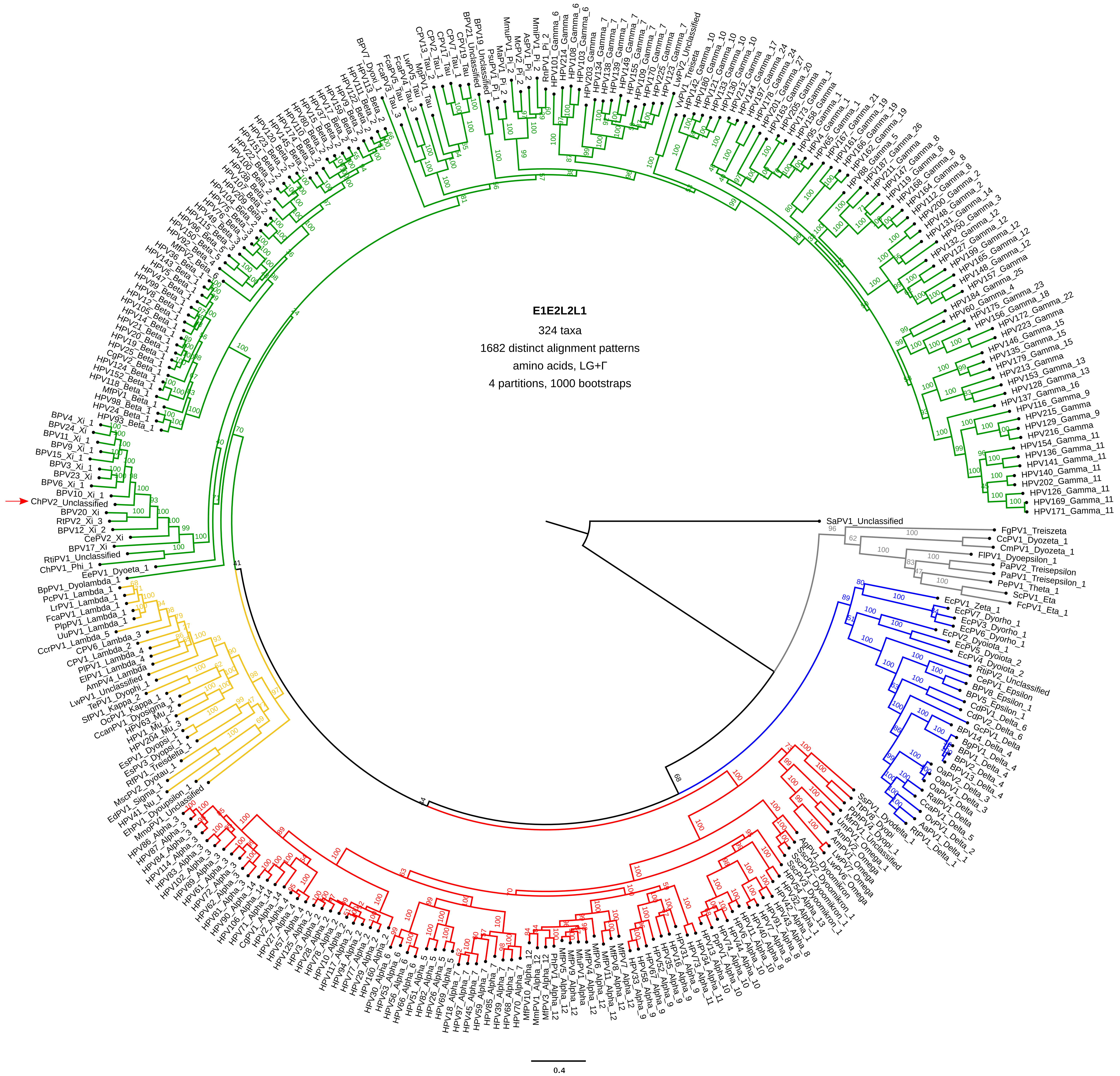

Figure S6. Maximum likelihood phylogenetic tree of the concatenated *E1E2L2L1* amino acid alignments of 324 PVs. Clade color codes highlight the different PV groups: red, Alpha-OmikronPVs; green, Beta-XiPVs; yellow, Lambda-MuPVs; blue, Delta-ZetaPVs; and gray, a yet unclassified clade consisting of PVs infecting birds and turtles. The position of ChPV2 is indicated with a red arrow. Values on branches correspond to ML bootstrap support values.

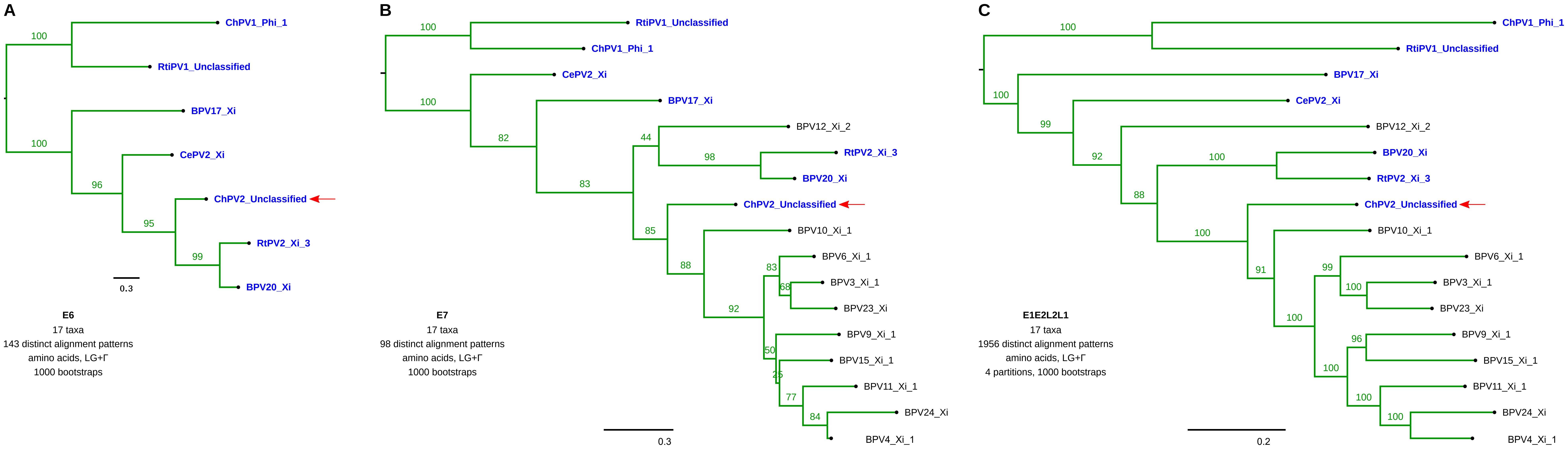

Figure S7. Maximum likelihood phylogenetic trees of *E6* (A), *E7* (B) and the concatenated *E1E2L2L1* (C) amino acid alignments of 17 PVs. The taxa highlighted in blue contain both the *E6* and *E7* oncogenes in their genomes, while the other taxa contain only *E7*. The position of ChPV2 is indicated with a red arrow. Values on branches correspond to ML bootstrap support values.

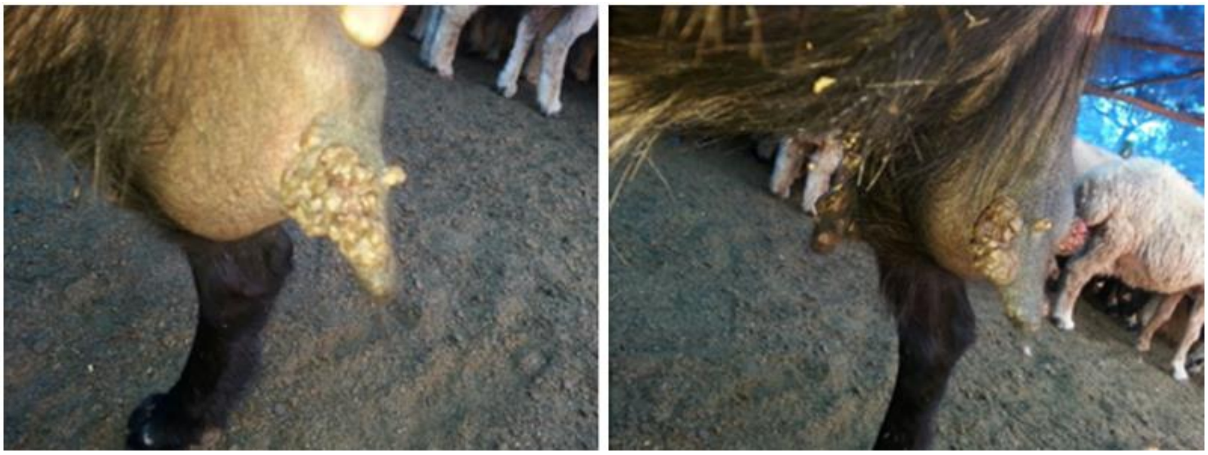

Figure S8: Macroscopic view of teat papilloma of a Damascus (Shami) goat taken from (12)

12: Dogan F, Dorttas SD, Bilge Dagalp S, Ataseven VS, Alkan F. A teat papillomatosis case in a Damascus goat (Shami goat) in Hatay province, Turkey: a new putative papillomavirus? Arch Virol. 2018 DOI: 10.1007/s00705-018-3781-2.
